# Asperindoles A–D and a *p*-Terphenyl Derivative from the Ascidian-Derived Fungus *Aspergillus* sp. KMM 4676

**DOI:** 10.3390/md16070232

**Published:** 2018-07-09

**Authors:** Elena V. Ivanets, Anton N. Yurchenko, Olga F. Smetanina, Anton B. Rasin, Olesya I. Zhuravleva, Mikhail V. Pivkin, Roman S. Popov, Gunhild von Amsberg, Shamil Sh. Afiyatullov, Sergey A. Dyshlovoy

**Affiliations:** 1 G.B. Elyakov Pacific Institute of Bioorganic Chemistry, Far Eastern Branch of the Russian Academy of Sciences, Prospect 100-letiya Vladivostoka, 159, Vladivostok 690022, Russia; ev.ivanets@yandex.ru (E.V.I.); smetof@rambler.ru (O.F.S.); abrus__54@mail.ru (A.B.R.); zhuravleva.oi@dvfu.ru (O.I.Z.); oid27@mail.ru (M.V.P.); prs_90@mail.ru (R.S.P.); afiyat@piboc.dvo.ru (S.S.A.); dyshlovoy@gmail.com (S.A.D.); 2 School of Natural Science, Far Eastern Federal University, Sukhanova St., 8, Vladivostok 690000, Russia; 3 Laboratory of Experimental Oncology, Department of Oncology, Hematology and Bone Marrow Transplantation with Section Pneumology, Hubertus Wald-Tumorzentrum, University Medical Center Hamburg-Eppendorf, 20246 Hamburg, Germany; g.von-amsberg@uke.de

**Keywords:** marine-derived fungi, secondary metabolites, indole-diterpenoids, cytotoxicity

## Abstract

Four new indole-diterpene alkaloids asperindoles A–D (**1**–**4**) and the known *p*-terphenyl derivative 3″-hydroxyterphenyllin (**5**) were isolated from the marine-derived strain of the fungus *Aspergillus* sp., associated with an unidentified colonial ascidian. The structures of **1**–**5** were established by 2D NMR and HRESIMS data. The absolute configurations of all stereocenters of **1**–**4** were determined by the combination of ROESY data, coupling constants analysis, and biogenetic considerations. Asperindoles C and D contain a 2-hydroxyisobutyric acid (2-HIBA) residue, rarely found in natural compounds. Asperindole A exhibits cytotoxic activity against hormone therapy-resistant PC-3 and 22Rv1, as well as hormone therapy-sensitive human prostate cancer cells, and induces apoptosis in these cells at low-micromolar concentrations.

## 1. Introduction

Marine fungi are promising and prolific sources of new biological active compounds. Fungi of the genus *Aspergillus*, section *Candidi* (*A. candidus*, *A. campestris*, *A. taichungensis*, *A. tritici*), are known to produce several types of *p*-terphenyl derivatives, such as terphenyllins (terphenyllin [1], 3-hydroxyterphenyllin [2], terprenins [3]) and candidusins (candidusins A–C [4], prenylcandidusins A–C [5]), and a number of flavonoid derivatives (e.g., chlorflavonin [6], chlorflavonin A [7]). These compounds exhibit antioxidant [8,9], cytotoxic [5,9,10], antimicrobial [9], and immunosuppressive activities [9]. Recently, several indole and pyrrolidine alkaloids unusual for this fungal group were isolated from a Thai sponge-derived *A. candidus* strain [11].

**Figure 1:**
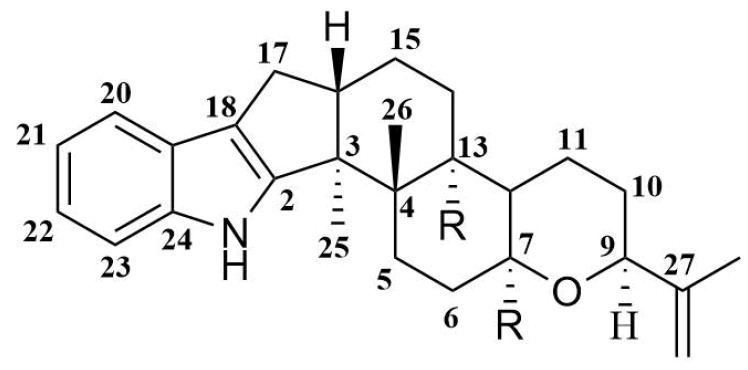
Usual framework of indole-diterpenes.

Indole-diterpene alkaloids are widely represented among the fungal metabolites. These compounds have been isolated from fungi of the genera *Claviceps*, *Acremonium*, *Eupenicillium*, *Penicillium*, and *Aspergillus* (including *Emericella striata*). Most natural indole-diterpenes have an invariable framework (Figure 1). Usually, C-7, C-13, and C-27 are oxidized. The oxygenation of C-27 is often followed by the formation of an ether bridge between C-27 and C-7 with inversion of the stereoconfiguration at C-7 [12]. Interestingly, some fungi produce metabolites with relatively rare features in the classical indole-diterpene backbone. For example, *Acremonium lolii* produces indoles diprenylated at C-20 and C-21, together with oxygenated derivatives [13]. Alkaloids with a 1,3-dioxane moiety joined at C-9 and C-10 with the F-ring from *A. lolii* have also been reported [14,15]. Many of such compounds showed tremorgenic [16], cytotoxic [17,18], and antiinsectan [19] activities, and some of them are antagonists of cannabinoid receptors [20].

Based on promising screening results in search of producers of biologically active compounds, the marine-derived fungus *Aspergillus* sp. KMM 4676, which is associated with an unidentified colonial ascidian (from the Shikotan Island in the Pacific Ocean), was selected for further studies. During earlier examinations of this fungal strain, five known *p*-terphenyls and one known flavonoid were isolated [21]. Herein, we describe the results of subsequent comprehensive chemical and bioactivity investigations of the extracts of strain KMM 4676, leading to the characterization of four new natural compounds.

## 2. Results

The HRESIMS spectrum of **1** exhibited a pseudo-molecular peak at *m*/*z* 526.1980 [M + H]^+^, showing the characteristic isotope pattern with one chlorine atom, therefore establishing its molecular formula as C_29_H_32_NO_6_Cl, which was supported by the ^13^C NMR spectrum.

Inspection of the ^1^H and ^13^C NMR data (Table 1, Figures S1–S2) of **1** revealed the presence of three quaternary methyls (*δ*_C_ 16.1, 17.0, 23.4; *δ*_H_ 1.02, 1.21, 1.31), one acetate methyl (*δ*_C_ 21.8, *δ*_H_ 2.07), six methylene *sp*^3^ (*δ*_C_ 20.8, 26.3, 26.9, 30.0, 31.5, 64.8; *δ*_H_ 1.66, 1.77, 1.91, 1.93, 2.00, 2.11, 2.30, 2.40, 2.55, 2.60, 3.67, 4.04), two methine *sp*^3^ (*δ*_C_ 48.3, 78.3, *δ*_H_ 2.72, 4.74), four methine *sp*^2^ (*δ*_C_ 111.3, 118.6, 118.7, 119.9; *δ*_H_ 6.11, 6.91, 7.26, 7.27), three quaternary oxygen-bearing *sp*^3^ (*δ*_C_ 75.0, 77.0, 93.6), two quaternary *sp*^3^ (*δ*_C_ 38.5, 51.4), and eight quaternary *sp*^2^ (*δ*_C_ 115.0, 123.3, 123.7, 140.2, 154.0, 159.1, 170.2, 195.9) carbons, as well as a NH singlet (*δ*_H_ 10.73) and an OH singlet (*δ*_H_ 5.10).

**Figure 2:**
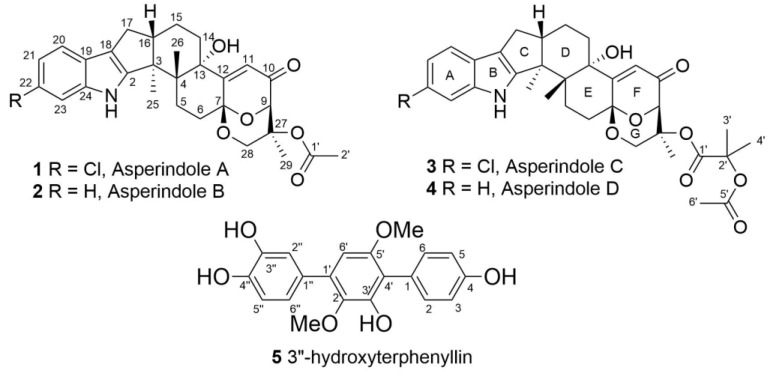
Chemical structures of **1**–**5**.

The ^1^H and ^13^C NMR spectra of **1** (Table 1) resembled those of paspalinine [16], suggesting that **1** has an indole-diterpene core similar to that of paspalinine. However, the differences in chemical shift values of C-19 (*δ*_C_ 123.3) and C-22 (*δ*_C_ 123.7) of **1** from the corresponding carbons in paspalinine [16]; the HMBC correlations (Figure 2, Figure S5) from H-20 (*δ*_H_ 7.26) to C-18 (*δ*_C_ 115.0) and C-22, from H-21 (*δ*_H_ 7.26) to C-19 and C-23 (*δ*_C_ 111.3), and from H-23 (*δ*_H_ 7.26) to C-19 and C-21 (*δ*_C_ 118.7); and the coupling constants *J*_H20–H21_ (8.5 Hz) and *J*_H21–H23_ (2.1 Hz) suggested the presence of a chlorine atom on C-22 of **1**.

**Figure 3:**
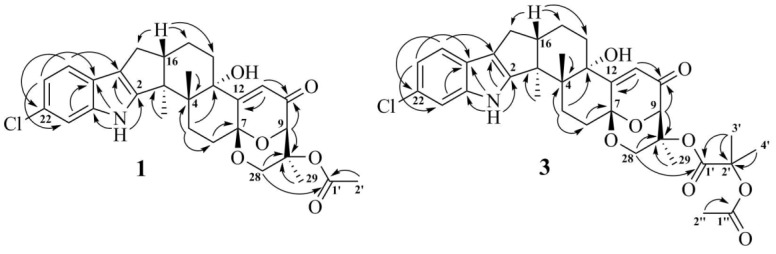
Key HMBC correlations of **1** and **3**.

**Figure 4:**
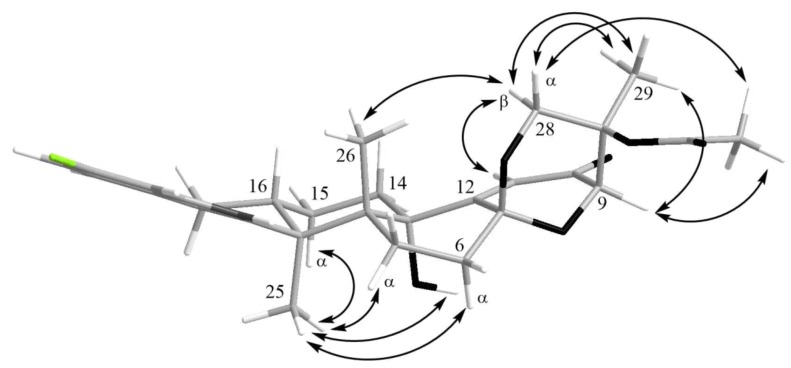
Key ROESY correlations in asperindole A (**1**).

**Figure 5:**
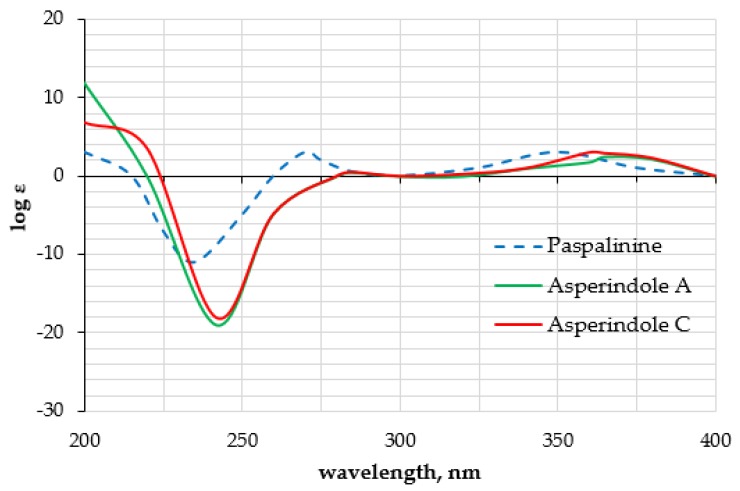
Experimental ECD data of **1**, **3**, and paspalinine [17].

The HMBC correlations (Figure 3, Figure S5) from H-28*β* (*δ*_H_ 3.67) to C-27 (*δ*_C_ 75.0), C-29 (*δ*_C_ 17.0), and C-1′ (*δ*_C_ 170.2); from H-9 (*δ*_H_ 4.74) to C-7 (*δ*_C_ 93.6), C-28 (*δ*_C_ 64.8), and C-27; from H-28*α* (*δ*_H_ 4.04) to C-7, C-9 (*δ*_C_ 78.3), and C-27; and from H_3_-29 (*δ*_H_ 1.21) to C-27 suggested the presence of a 1,3-dioxane ring with an acetoxy group at C-27. The W-type coupling constant *J*_H9–H28*α*_ (2.5 Hz) and ROESY correlations (Figure 4, Figure S6) of H-28*β* with H-11 (*δ*_H_ 6.11), H_3_-29, H_3_-26 (*δ*_H_ 1.02), and of H-28*α* with H_3_-29 indicated a relative configuration of chiral centers in the 1,3-dioxane ring as 7R*, 9R*, 27S*. The ROESY correlations (Figure 4, Figure S6) of H_3_-25 (*δ*_H_ 1.31) with H-5*α* (*δ*_H_ 1.93), 13-OH (*δ*_H_ 5.10), H-6*α* (*δ*_H_ 2.00), and H-15*α* (*δ*_H_ 1.91), and of H-16 (*δ*_H_ 2.72) with H_3_-26 (*δ*_H_ 1.02) suggested the relative configurations of the stereogenic carbons of the C–G rings in **1** as 3S*, 4R*, 13S*, 16R*. The absolute configurations of all stereocentres in **1** was proposed as 3*S*, 4*R*, 7*R*, 9*R*, 13*S*, 16*R*, 27*S*—the same as those in paspalinine—based on biosynthetic considerations, and was confirmed by the comparison of the ECD (electronic circular dichroism) spectral data with that of paspalinine [17] (Figure 5). Compound **1** was named asperindole A. It should be noted that chlorinated indolediterpenes are rare in nature [12,22,23,24,25].

The molecular formula of **2** was determined as C_29_H_33_NO_6_ by a HRESIMS peak at *m*/*z* 490.2188 [M − H]^−^, which was supported by the ^13^C NMR spectrum. The general features of the ^1^H and ^13^C NMR spectra (Table 1, Figures S7 and S8) of **2** resemble those of **1**, with the exception of the proton and carbon signals of an indole moiety, as well as the absence of a chlorine atom as evidenced by the HRESIMS spectrum. The coupling constants and the multiplicity of the aromatic protons in ring A (H-20, *δ*_H_ 7.25, d, *J* = 7.6 Hz; H-21, *δ*_H_ 6.89, t, *J* = 7.6 Hz; H-22, *δ*_H_ 6.93, t, *J* = 7.6 Hz; and H-23, *δ*_H_ 7.27, d, *J* = 7.6 Hz) allowed the conclusion to be made that **2** is a nonchlorinated analogue of **1**. Compound **2** was therefore named asperindole B.

The molecular formula of **3** was established as C_33_H_38_NO_8_Cl on the basis of the HRESIMS, containing a peak at *m*/*z* 610.2206 [M − H]^–^, and was supported by the ^13^C NMR spectrum. The analysis of the NMR data (Figures S14–S20) for **3** revealed the presence of the same indole-diterpene framework as that in **1**, with the exception of the proton and carbon signals in a 1,3-dioxane ring, as well as the presence of two methyl (*δ*_C_ 23.9, 24.2), an ester carbonyl (*δ*_C_ 171.1), and an oxygen-bearing quaternary *sp*^3^ (*δ*_C_ 77.9) carbons. The HMBC correlations (Figure 3, Figure S19) from H-3′ (*δ*_H_ 1.52) and H-4′ (*δ*_H_ 1.54) to C-2′ (*δ*_C_ 77.9), and from H-6′ (*δ*_H_ 2.04) to C-5′ (*δ*_C_ 169.3) suggested the presence of an acetylated residue of 2-hydroxyisobutyric acid (2-HIBA) in **3**. This was corroborated by the molecular weight of **3**, which was 86 amu (C_4_H_6_O_2_) greater than that of **1**. The ROESY correlations of **3** (Figure S20) were similar to those in **1** (Figure 4, Figure S6). Based on these data and together with the ECD spectrum of **1** (Figure 5), the absolute configurations of all stereocentres in **3** were proposed to be the same as those in asperindole A. Consequently, **3** was named asperindole C. To the best of our knowledge, the 2-HIBA residue is unique amongst naturally occurring compounds.

The HRESIMS spectrum of **4** exhibited the [M − H]^–^ peak at *m*/*z* 576.2594, corresponding to C_33_H_39_NO_8_, which was supported by the ^13^C NMR spectrum. The general features of the ^1^H and ^13^C NMR spectra (Table 2, Figures S21 and S22) of **4** resembled those of **3**, with the exception of some proton and carbon signals of the indole moiety. Similar to **2**, the coupling constants and multiplicity of the aromatic protons in ring A (H-20, *δ*_H_ 7.25, d, *J* = 7.5 Hz; H-21, *δ*_H_ 6.88, brt, *J* = 7.2 Hz; H-22, *δ*_H_ 6.92, brt, *J* = 7.1 Hz; and H-23, *δ*_H_ 7.27, d, *J* = 6.9 Hz) led to the conclusion that **4** is a nonchlorinated analogue of **3**. Compound **4** was therefore named asperindole D.

**Figure 6:**
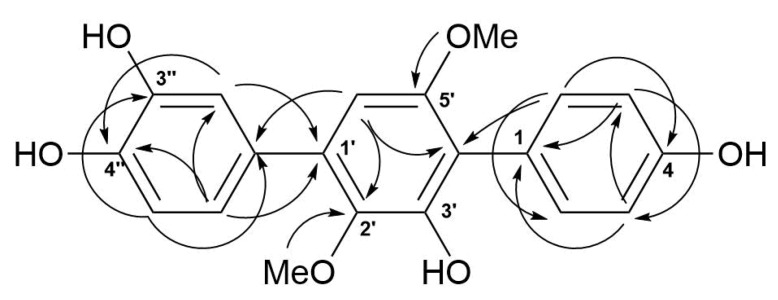
Key HMBC correlations of **5**.

The molecular formula of **5** was determined as C_20_H_18_O_6_, based on a pseudo-molecular peak at *m*/*z* 353.1013 [M − H]^−^ from the HRESIMS spectrum. This was supported by the ^13^C NMR spectrum. A close inspection of the ^1^H and ^13^C NMR data (Table 3, Figures S23 and S24) of **5** revealed the presence of eight aromatic protons (*δ*_H_ 6.47, 6.85, 2H; 6.91, 7.02, 7.19, 7.25, 2H) and eight methine *sp*^2^ (*δ*_C_ 104.8, 115.9, 115.9, 116.8, 117.5, 122.0, 133.7, 133.7), six oxygen-bearing quaternary *sp*^2^ (*δ*_C_ 140.8, 146.3, 146.4, 149.8, 155.1, 157.6), and four quaternary *sp*^2^ (*δ*_C_ 118.3, 126.8, 131.9, 134.2) carbons, and two methoxy groups (*δ*_C_ 56.8, 61.4; *δ*_H_ 3.41, 3.71). A direct comparison of ^1^H and ^13^C NMR spectra of **5** (Table 3, Figures S23 and S24) with those of terphenyllin [1] showed their close resemblance, with the exception of the presence of a hydroxy group at C-3″ (*δ*_C_ 146.4) and the difference in carbon chemical shifts at C-2″ (*δ*_C_ 117.5), C-4″ (*δ*_C_ 146.3), C-5″ (*δ*_C_ 116.8) and C-6″ (*δ*_C_ 122.0) (131.1 ppm for C-1″, C-2″, and C-6″; 158.0 ppm for C-4″ in terphenyllin [1]). The HMBC correlations (Figure 6, Figure S27) from H-2 (*δ*_H_ 7.25) to C-4 (*δ*_C_ 157.6), C-6 (*δ*_C_ 133.7), and C-4′ (*δ*_C_ 118.3); from H-3 (*δ*_H_ 6.85) to C-1 (*δ*_C_ 126.8) and C-5 (*δ*_C_ 115.9); from H-5 (*δ*_H_ 6.85) to C-3 (*δ*_C_ 115.9) and C-1; from H-6 (*δ*_H_ 7.25) to C-2 (*δ*_C_ 133.7), C-4, and C-4′; from H-6′ (*δ*_H_ 6.47) to C-2′ (*δ*_C_ 140.8), C-4′, and C-1″ (*δ*_C_ 131.9); from H-2″ (*δ*_H_ 7.19) to C-1′ (*δ*_C_ 134.2), C-4″, and C-6″; from H-5″ (*δ*_H_ 6.91) to C-1″ and C-3″; from H-6″ (*δ*_H_ 7.50) to С-1′, C-2″, and C-4″; and ROESY correlation from H-6′ and 2′-OMe (*δ*_H_ 3.41) to H-2″ and H-6″, and from 5′-OMe (*δ*_H_ 3.71) to H-2 (H-6) established the structure of **5** as the 2′,5′-dimethoxy-4,3′,3″,4″-tetrahydroxy-*p*-terphenyl derivative. This structure was previously published as hydroxyterphenyllin in an unavailable source [26]. It should be noted that these authors reported the name “hydroxyterphenyllin” for an isomeric compound [27] now known as 3-hydroxyterphenyllin [2,28]. Probably, the structure of **5** was mistakenly provided by [26] instead of the structure of 3-hydroxyterphenyllin. Therefore, **5** should be named 3″-hydroxyterphenyllin.

**Figure 7:**
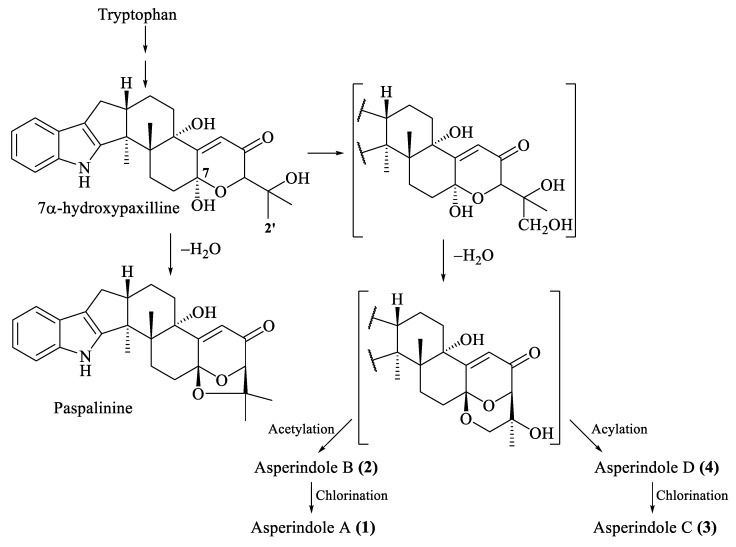
Proposed biosynthesis of asperindoles A–D (**1**–**4**).

The biosynthesis of related indole-diterpenes was previously proposed for paspalinine [29]. Apparently, the common biosynthetic precursor of asperindoles and 1,3-dioxolane indole-diterpenoids (including paspalinine) is 7*α*-hydroxypaxilline (Figure 7). Oxidation of the isopropyl substituent, followed by cyclization at С-7 and С-2′, generates a 1,3-dioxane ring. Asperindoles are then formed by acylation and halogenation. 

**Figure 8:**
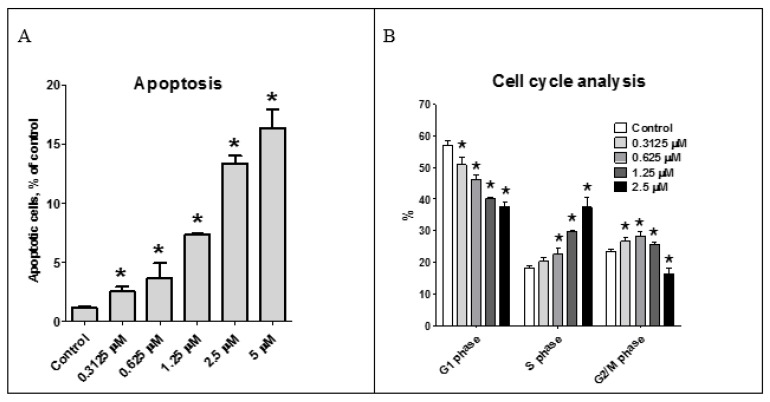
Effect of asperindole A (**1**) on cell cycle progression and apoptosis induction. Apoptotic cells were detected as a sub-G1 population (**A**); Cell cycle analysis of 22Rv1 cells treated with asperindole A (**1**) for 48 h (**B**). Cell cycle phase distribution, quantified using the Cell Quest Pro software. * *p* < 0.05.

The effect of the asperindoles A (**1**) and C (**3**) on cell viability, cell cycle progression, and induction of apoptosis in human prostate cancer cell lines was investigated. MTT assays revealed that asperindole C (**3**) was noncytotoxic against human PC-3, LNCaP (androgen-sensitive human prostate adenocarcinoma cells), and 22Rv1 cell lines with an IC_50_ > 100 µM. In contrast, asperindole A (**1**) showed cytotoxicity in all three cell lines, with IC_50_ values of 69.4 µM, 47.8 µM, and 4.86 µM, respectively. Docetaxel, which was used as a reference substance, displayed IC_50_ values of 15.4 nM, 3.8 nM, and 12.7 nM, respectively. Asperindole A (**1**) was able to induce apoptosis in human cancer 22Rv1 cells at low-micromolar concentrations (Figure 8). Cell cycle progression analysis of 22Rv1 cells treated with asperindole A (**1**) for 48 h revealed a S-phase arrest (as well as a discrete G2/M-phase arrest, Figure 8). Thus, asperindole A (**1**) may be a promising candidate for further studies in human drug-resistant prostate cancer. In contrast, 22Rv1 cells treated with 100 µM of asperindole C (**3**) for 48 h revealed only minimal induction of apoptosis (8.9 ± 0.6% vs 1.2 ± 0.1% in the control) and no significant changes in cell cycle progression.

## 3. Materials and Methods

### 3.1. General Experimental Procedures

Optical rotations were measured on a Perkin-Elmer 343 polarimeter (Perkin Elmer, Waltham, MA, USA). UV spectra were recorded on a Specord UV−vis spectrometer (Carl Zeiss, Jena, Germany) in CHCl_3_. NMR spectra were recorded in DMSO-*d*_6_ on a Bruker DPX-500 (Bruker BioSpin GmbH, Rheinstetten, Germany) and a Bruker DRX-700 (Bruker BioSpin GmbH, Rheinstetten, Germany) spectrometer, using TMS as an internal standard. HRESIMS spectra were measured on an Agilent 6510 Q-TOF LC mass spectrometer (Agilent Technologies, Santa Clara, CA, USA) and a Maxis impact mass spectrometer (Bruker Daltonics GmbH, Rheinstetten, Germany).

Low-pressure liquid column chromatography was performed using silica gel (50/100 μm, Imid, Russia). Plates (4.5 cm × 6.0 cm) precoated with silica gel (5–17 μm, Imid) were used for thin-layer chromatography. Preparative HPLC was carried out on a Shimadzu LC-20 chromatograph (Shimadzu USA Manufacturing, Canby, OR, USA) using a YMC ODS-AM (YMC Co., Ishikawa, Japan) (5 µm, 10 mm × 250 mm) and YMC SIL (YMC Co., Ishikawa, Japan) (5 µm, 10 mm × 250 mm) columns with a Shimadzu RID-20A refractometer (Shimadzu Corporation, Kyoto, Japan).

### 3.2. Fungal Strain

The strain was isolated from an unidentified colonial ascidian (Shikotan Island, Pacific Ocean) on malt extract agar, and identified on the basis of morphological and molecular features. For DNA extraction, the culture was grown on malt extract agar under 25 °C for 7 days. DNA extraction was performed with the HiPurATM Plant DNA Isolation kit (CTAB Method) (HiMedia Laboratories Pvt. Ltd., Mumbai, India) according to the manufacturer’s instructions. Fragments containing the ITS (internal transcribed spacer) regions were amplified using ITS1 and ITS4 primers. The newly obtained sequences were checked visually and compared to available sequences in the GenBank database (www.mycobank.org). According to BLAST analysis of the ITS1–5.8S–ITS2 sequence, the strain KMM 4676 had 98% similarity with *Aspergillus candidus.* The sequences were deposited in the GenBank nucleotide sequence database under MG 241226. The strain is deposited in the Collection of Marine Microorganisms of G. B. Elyakov Pacific Institute of Bioorganic Chemistry FEB RAS under the code KMM 4676.

### 3.3. Cultivation of Fungus

The fungus was cultured at 22 °С for three weeks in 14 × 500 mL Erlenmeyer flasks, each containing rice (20.0 g), yeast extract (20.0 mg), KH_2_PO_4_ (10 mg), and natural sea water (40 mL).

### 3.4. Extraction and Isolation

The fungal mycelia with the medium were extracted for 24 h with 5.6 L of EtOAc. Evaporation of the solvent under reduced pressure gave a dark brown oil (6.25 g), to which 250 mL H_2_O–EtOH (4:1) was added, and the mixture was thoroughly stirred to yield a suspension. It was extracted successively with *n*-hexane (150 mL × 2), EtOAc (150 mL × 2), and *n*-BuOH (150 mL × 2). After evaporation of the EtOAc layer, the residual material (3.92 g) was passed over a silica gel column (35.0 cm × 2.5 cm, 75 g), which was eluted first with *n*-hexane (1.0 L), followed by a step gradient from 5% to 100% EtOAc in *n*-hexane (total volume 30 L). Fractions of 250 mL each were collected and combined on the basis of TLC (Si gel, toluene–2-propanol, 6:1 and 3:1, *v*/*v*).

The *n*-hexane–EtOAc (9:1, 2 L, 21.70 mg) fraction was purified by LH-20 column (80 cm × 2 cm, 50 g) with CHCI_3_ to yield 30 subfractions. Subfraction 6 is the individual compound **1** (8.30 mg). Subfractions 8–12 (7.00 mg) were purified by HPLC on a YMC ODS-AM column, eluting with MeOH–H_2_O (9:1), and then by HPLC on a YMC SIL column, eluting with acetone–*n*-hexane (1:3) to yield **2** (0.56 mg), **3** (1.05 mg), and **4** (1.47 mg). The *n*-hexane–EtOAc (4:1, 2 L, 145 mg) fraction was purified by by HPLC on a YMC ODS-AM column, eluting with MeOH–H_2_O (13:7), then by HPLC on a YMC-SIL column eluting first with CHCl_3_–MeOH–NH_4_OAc (90:10:1.5), and then with CHCl_3_–MeOH–NH_4_OAc (85:15:1) to yield **5** (56.10 mg).

Asperindole A (**1**): white powder; [*α*${]}_{D}^{20}$ +22 (*c* 0.10, CHCl_3_); UV (MeOH) *λ*_max_ (log *ε*) 284 (3.86), 236.4 (4.55), 195.6 (4.55) nm; ECD (0.21 mM, MeOH) *λ*_max_ (Δ*ε*) 205 (+8.52), 240 (−19.80), 280 (+0.25), 360 (+3.60) nm; ^1^H and ^13^C NMR data see Table 1, Figures S1–S6; HR ESIMS *m*/*z* 526.1980 [M + H]^+^ (calcd. for C_29_H_33_NO_6_Cl, 526.1992, Δ −2.28 ppm).

Asperindole B (**2**): white powder; [*α*${]}_{D}^{20}$ +40 (*c* 0.03, CHCl_3_); ^1^H and ^13^C NMR data see Table 1, Figures S7–S13; HRESIMS *m*/*z* 514.2194 [M + Na]^+^ (calcd. for C_29_H_33_NO_6_Na, 514.2200, Δ −1.17 ppm).

Asperindole C (**3**): white powder; [*α*${]}_{D}^{20}$ +46 (*c* 0.72, CHCl_3_); UV (MeOH) *λ*_max_ (log *ε*) 284 (3.90), 236.4 (4.56), 194.8 (4.46) nm; ECD (0.21 mM, MeOH) *λ*_max_ (Δ*ε*) 205 (+6.43), 240 (−18.25), 280 (+0.02), 360 (+3.57) nm; ^1^H and ^13^C NMR data see Table 2, Figures S14–S20; HRESIMS *m*/*z* 610.2206 [M − H]^–^ (calcd. for C_33_H_37_NO_8_Cl, 610.2213, Δ −1.15 ppm).

Asperindole D (**4**): white powder; [*α*${]}_{D}^{20}$ +24 (*c* 0.05, CHCl_3_); ^1^H and ^13^C NMR data, see Table 2, Figures S21 and S22; HRESIMS *m*/*z* 576.2594 [M − H]^–^ (calcd. for C_33_H_38_NO_8_, 576.2603, Δ −1.56 ppm).

3″-hydroxyterphenylline (**5**)*:* ^1^H and ^13^C NMR data, see Table 3, Figures S23–S27; HRESIMS *m*/*z* 353.1036 [M − H]^–^ (calcd. for C_20_H_17_O_6_, 353.1031, Δ −1.42 ppm).

### 3.5. Cell Culture

The human prostate cancer cells lines 22Rv1, PC-3, and LNCaP were purchased from ATCC. Cell lines were cultured in 10% FBS/RPMI media (Invitrogen Ltd., Paisley, UK) with (for LNCaP) or without (for 22Rv1 and PC-3) 1 mM sodium pyruvate (Invitrogen). Cells were continuously kept in culture for a maximum of 3 months, and were routinely inspected microscopically for stable phenotype and regularly checked for contamination with mycoplasma. Cell line authentication was performed by DSMZ (Braunschweig, Germany) using highly polymorphic short tandem repeat loci [30].

### 3.6. Cytotoxicity Assay

The in vitro cytotoxicity of individual substances was evaluated using the MTT (3-(4,5-dimethylthiazol-2-yl)-2,5-diphenyltetrazolium bromide) assay, which was performed as previously described [31]. Docetaxel was used as a control.

### 3.7. Cell Cycle and Apoptosis Induction Analysis

The cell cycle distribution was analyzed by flow cytometry using PI (propidium iodide) staining as described before with slight modifications [32]. In brief, cells were preincubated overnight in 6-well plates (2 × 10^5^ cells/well in 2 mL/well). The medium was changed to fresh medium containing different concentrations of the substances. After 48 h of treatment, cells were harvested with a trypsin-EDTA solution, fixed with 70% EtOH, stained, and analyzed by BD Bioscience FACS Calibur analyzer (BD Bioscience, Bedford, MA, USA). The results were quantitatively analyzed using BD Bioscience Cell Quest Pro v.5.2.1. software (San Jose, CA, USA). Cells detected in the sub-G1 peak were considered as apoptotic.

## 4. Conclusions

Four new metabolites, the indole-diterpene alkaloids asperindoles A–D (**1**–**4**), and the known *p*-terphenyl derivative 3′′-hydroxyterphenyllin (**5**) were isolated from a marine-derived strain of the fungus *A. candidus* KMM 4676, associated with an unidentified colonial ascidian. To the best of our knowledge, **3** and **4** are the first examples of naturally occurring compounds containing a 2-hydroxyisobutiric acid (2-HIBA) residue. This is the first report of the spectral data and reliable assignment for 3′′-hydoxyterphenyllin (**5**). Asperindole A (**1**) was proved to be highly cytotoxic in 22Rv1 human prostate cancer cells resistant to androgen receptor-targeted therapies. Therefore, this compound is a promising candidate for further evaluation in human drug-resistant prostate cancer cells. 

## Figures and Tables

**Table 1:** ^13^С NMR data (125 MHz, *δ* in ppm, DMSO-*d*_6_) for asperindoles A–D (**1**–**4**).

Position	1	2	3	4
2	154.0, C	152.8, C	154.1, C	152.8, C
3	51.4, C	51.2, C	51.4, C	51.2, C
4	38.5, C	38.6, C	38.5, C	38.6, C
5	26.3, CH_2_	26.2, CH_2_	26.3, CH_2_	26.2, CH_2_
6	30.0, CH_2_	30.1, CH_2_	30.0, CH_2_	30.0, CH_2_
7	93.6, C	93.6, C	93.6, C	93.5, C
9	78.3, CH	78.3, CH	78.3, CH	78.6, CH
10	195.9, C	195.9, C	195.9, C	195.7, C
11	119.9, CH	119.9, CH	119.9, CH	119.8, CH
12	159.1, C	159.2, C	159.1, C	159.2, C
13	77.0, C	77.0, C	77.0, C	77.0, C
14	31.5, CH_2_	31.6, CH_2_	31.5, CH_2_	31.5, CH_2_
15	20.8, CH_2_	20.9, CH_2_	20.82, CH_2_	20.9, CH_2_
16	48.3, CH	48.3, CH	48.3, CH	48.4, CH
17	26.9, CH_2_	27.1, CH_2_	26.9, CH_2_	27.1, CH_2_
18	115.0, C	114.8, C	115.0, C	114.8, C
19	123.3, C	124.6, C	123.3, C	124.6, C
20	118.6, CH	117.5, CH	118.6, CH	117.5, CH
21	118.7, CH	118.3, CH	118.7, CH	118.3, CH
22	123.7, C	119.1, CH	123.7, C	119.1, CH
23	111.3, CH	111.8, CH	111.3, CH	111.8, CH
24	140.2, C	139.9, C	140.2, C	139.9, C
25	16.1, CH_3_	16.2, CH_3_	16.1, CH_3_	16.2, CH_3_
26	23.4, CH_3_	23.4, CH_3_	23.4, CH_3_	23.3, CH_3_
27	75.0, C	75.0, C	75.8, C	75.8, C
28	64.8, CH_2_	64.8, CH_2_	64.1, CH_2_	64.1, CH_2_
29	17.0, CH_3_	17.0, CH_3_	16.4, CH_3_	16.4, CH_3_
1′	170.2, C	170.2, C	171.1, C	171.1, C
2′	21.8, CH_3_	21.8, CH_3_	77.9, C	77.9, C
3′			23.9, CH_3_	23.9, CH_3_
4′			24.2, CH_3_	24.2, CH_3_
1″			169.3, C	169.3, C
2″			20.75, CH_3_	20.8, CH_3_

**Table 2:** ^1^H NMR data (*δ* in ppm, *J* in Hz, DMSO-*d*_6_) for asperindoles A–D (**1**–**4**).

Position	1 *	2 **	3 **	4 **
NH	10.73, brs	10.54, brs	10.73, s	10.52, s
5*α* 5*β*	1.93, m 2.40, dd (13.4, 10.0)	1.96, m 2.41, t (12.3)	1.95, m 2.39, dd (13.4,10.0)	1.96, m 2.41, t (12.3)
6*α* 6*β*	2.00, dd (12.9, 8.6) 2.55, m	1.99, m 2.55, m	1.95, dd (12.9, 8.6) 2.55, m	1.95, dd (12.9, 8.6) 2.55, m
9	4.74, d (2.3)	4.74, d (2.1)	4.74, d (2.3)	4.63, d (2.4)
11	6.11, s	6.11, s	6.11, s	6.12, s
14*α* 14*β*	2.11, dt (13.6, 2.8) 1.77, td (13.2, 4.5)	2.12, brd (13.4) 1.78, brt (13.4)	2.12, dt (13.6, 2.8) 1.76, td (13.2, 4.5)	
15*α* 15*β*	1.91, m 1.66, m	1.91, m 1.66, m	1.91, m 1.65, m	1.91, m 1.65, m
16	2.72, m	2.72, m	2.72, m	2.63, m
17*α* 17*β*	2.30, dd (13.0, 10.9) 2.60, dd (13.0, 6.4)	2.30, t (12.3) 2.60, dd (12.3, 6.6)	2.30, dd (13.0, 10.9) 2.60, dd (13.0, 6.4)	2.31, dd (13.0, 10.9) 2.60, dd (13.0, 6.2)
20	7.26, d (8.6)	7.25, d (7.6)	7.26, d (8.3)	7.25, d (7.5)
21	6.91, dd (8.3, 1.9)	6.89, t (7.6)	6.91, dd (8.3, 2.0)	6.88, brt (7.2)
22		6.93, t (7.6)		6.92, brt (7.1)
23	7.27, d (2.2)	7.27, d (7.6)	7.25, d (2.0)	7.27, d (6.9)
25	1.31, s	1.30, s	1.31, s	1.30, s
26	1.02, s	1.03, s	1.02, s	1.03, s
28*α* 28*β*	4.04, dd (13.4, 2.5) 3.67, d (13.4)	4.05, dd (13.3, 2.1) 3.68, d (13.3)	4.11, dd (13.2, 2.5) 3.68, d (13.2)	4.11, dd (13.4, 2.4) 3.69, d (13.4)
29	1.21, s	1.21, s	1.17, s	1.18, s
3′			1.52, s	1.52, s
4′			1.54, s	1.54, s
2″	2.07, s	2.07, s	2.04, s	2.04, s
13-OH	5.10, s	5.08, s	5.11, s	5.08, s

^1^H NMR spectroscopic data were measured at * 700 MHz and ** 500 MHz, respectively.

**Table 3:** ^1^H and ^13^С NMR data (*δ* in ppm, DMSO-*d*_6_) for 3″-hydroxyterphenyllin (**5**).

Position	*δ*_С_, mult	*δ*_H_ (*J* in Hz)	HMBC	ROESY
1	126.8, C			
2	133.7, CH	7.25, d (8.4)	4, 6, 4′	
3	115.9, CH	6.85, d (8.5)	1, 5	
4	157.6, C			
5	115.9, CH	6.85, d (8.5)	1, 3	
6	133.7, CH	7.25, d (8.4)	2, 4, 4′	5′-OMe
1′	134.2, C			
2′	140.8, C			
3′	149.8, C			
4′	118.3, C			
5′	155.1, C			
6′	104.8, CH	6.47, s	2′, 4′, 1″	2″, 6″
1″	131.9, C			
2″	117.5, CH	7.19, d (2.1)	1′, 4″, 6″	6′, 2′-OMe
3″	146.4, C			
4″	146.3, C			
5″	116.8, CH	6.91, d (8.1)	1″, 3″	
6″	122.0, CH	7.02, dd (8.1, 2.1)	1′, 2″, 4″	6′, 2′-OMe
2′-OMe	61.4, CH_3_	3.41, s	2′	2″, 6”
5′-OMe	56.8, CH_3_	3.71, s	5′	6

^1^H NMR and ^13^C NMR spectroscopic data were measured at 700 MHz and 175 MHz, respectively.

## Supplementary Materials

^1^H, ^13^C, DEPT, COSY-45, HSQC, HMBC, and ROESY spectra of new compounds **1**–**5** are available online at http://www.mdpi.com/1660-3397/16/7/232/s1.
