# Supplementary material for: Asperindoles A–D and a p-Terphenyl Derivative from the Ascidian-Derived Fungus Aspergillus sp. KMM 4676
Source: Mar Drugs. 2018 Jul 9;16(7):232. doi: 10.3390/md16070232 (PMC6070833; doi:10.3390/md16070232)
Supplement: Supplementary file 1 [file marinedrugs-16-00232-s001.pdf]

Supplementary data

Asperindoles A-D and *p*-Terphenyl Derivative from the Ascidian-Derived Fungus *Aspergillus candidus* Link KMM 4676

Elena V. Ivanets [1], Anton N. Yurchenko [1], Olga F. Smetanina [1], Anton B. Rasin [1], Olesya I. Zhuravleva[1,2], Mikhail V. Pivkin [1], Roman S. Popov [1], Gunhild von Amsberg [3], Shamil Sh. Afiyatullov [1] and Sergey A. Dyshlovoy [1,2,3]

[1] G.B. Elyakov Pacific Institute of Bioorganic Chemistry, Far Eastern Branch of the Russian Academy of Sciences, Prospect 100-letiya Vladivostoka, 159, Vladivostok 690022, Russia; ev.ivanets@yandex.ru (E.I.), yurchant@ya.ru (A.Y.); smetof@rambler.ru (O.S.); abrus__54@mail.ru (A.R.); oid27@mail.ru (M.P); prs_90@mail.ru (R.P.); afiyat@piboc.dvo.ru (S.A.)

[2] School of Natural Science, Far Eastern Federal University, Sukhanova St., 8, Vladivostok 690000, Russia;

[3] Laboratory of Experimental Oncology, Department of Oncology, Hematology and Bone Marrow Transplantation with Section Pneumology, Hubertus Wald-Tumorzentrum, University Medical Center Hamburg-Eppendorf, Hamburg 20246, Germany; cbokemeyer@uke.de (C.B.); g.von-amsberg@uke.de (G.A.); dyshlovoy@gmail.com (S.D.)

* Correspondence: yurchant@ya.ru; Tel.: +7-423-231-1168

Mar. Drugs 2018, 16, 232; doi:10.3390/md16070232 www.mdpi.com/journal/marinedrugs

Content

Figure S1. ^{1}H NMR (700 MHz, DMSO-d_6) spectrum of asperindole A (**1**)....................................3

Figure S2. ^{13}C NMR (125 MHz, DMSO-d_6) spectrum of asperindole A (**1**) 5

Figure S3. DEPT-135 (125 MHz, DMSO-d_6) spectrum of asperindole A (**1**) 6

Figure S4. HSQC (700 MHz, DMSO-d_6) spectrum of asperindole A (**1**)..7

Figure S5. HMBC (700 MHz, DMSO-d_6) spectrum of asperindole A (**1**)..8

Figure S6. ROESY (700 MHz, DMSO-d_6) spectrum of asperindole A (**1**) 9

Figure S7. ^{1}H NMR (500 MHz, DMSO-d_6) spectrum of asperindole B (**2**)..................................10

Figure S8. ^{13}C NMR (125 MHz, DMSO-d_6) spectrum of asperindole B (**2**)...............................12

Figure S9. DEPT-135 (125 MHz, DMSO-d_6) spectrum of asperindole B (**2**) 13

Figure S10. ^{1}H-^{1}H COSY (500 MHz, DMSO-d_6) spectrum of asperindole B (**2**)........................14

Figure S11. HSQC (500 MHz, DMSO-d_6) spectrum of asperindole B (**2**) 15

Figure S12. HMBC (500 MHz, DMSO-d_6) spectrum of asperindole B (**2**) 16

Figure S13. ROESY (500 MHz, DMSO-d_6) spectrum of asperindole B (**2**)...............................17

Figure S14. ^{1}H NMR (500 MHz, DMSO-d_6) spectrum of asperindole C (**3**) 18

Figure S15. ^{13}C NMR (125 MHz, DMSO-d_6) spectrum of asperindole C (**3**)..............................20

Figure S16. DEPT-135 (125 MHz, DMSO-d_6) spectrum of asperindole C (**3**) 21

Figure S17. ^{1}H-^{1}H COSY (500 MHz, DMSO-d_6) spectrum of asperindole C (**3**)........................22

Figure S18. HSQC (500 MHz, DMSO-d_6) spectrum of asperindole C (**3**) 23

Figure S19. HMBC (500 MHz, DMSO-d_6) spectrum of asperindole C (**3**) 24

Figure S20. ROESY (500 MHz, DMSO-d_6) spectrum of asperindole C (**3**)................................25

Figure S21. ^{1}H NMR (500 MHz, DMSO-d_6) spectrum of asperindole D (**4**) 26

Figure S22. ^{13}C NMR (125 MHz, DMSO-d_6) spectrum of asperindole D (**4**) 27

Figure S23. ^{1}H NMR (700 MHz, acetone-d_6) spectrum of 3″-hydroxyterphenyllin (**5**)...............28

Figure S24. ^{13}C NMR (125 MHz, acetone-d_6) spectrum of 3″-hydroxyterphenyllin (**5**) 29

Figure S25. DEPT-135 (125 MHz, acetone-d_6) spectrum of 3″-hydroxyterphenyllin (**5**) 30

Figure S26. HSQC (700 MHz, acetone-d_6) spectrum of 3″-hydroxyterphenyllin (**5**)..................31

Figure S27. HMBC (700 MHz, acetone-d_6) spectrum of 3″-hydroxyterphenyllin (**5**).................32

Figure S28. ROESY (700 MHz, acetone-d_6) spectrum of 3″-hydroxyterphenyllin (**5**) 33

Figure S1. ^{1}H NMR (700 MHz, DMSO-d_6) spectrum of asperindole A (**1**)

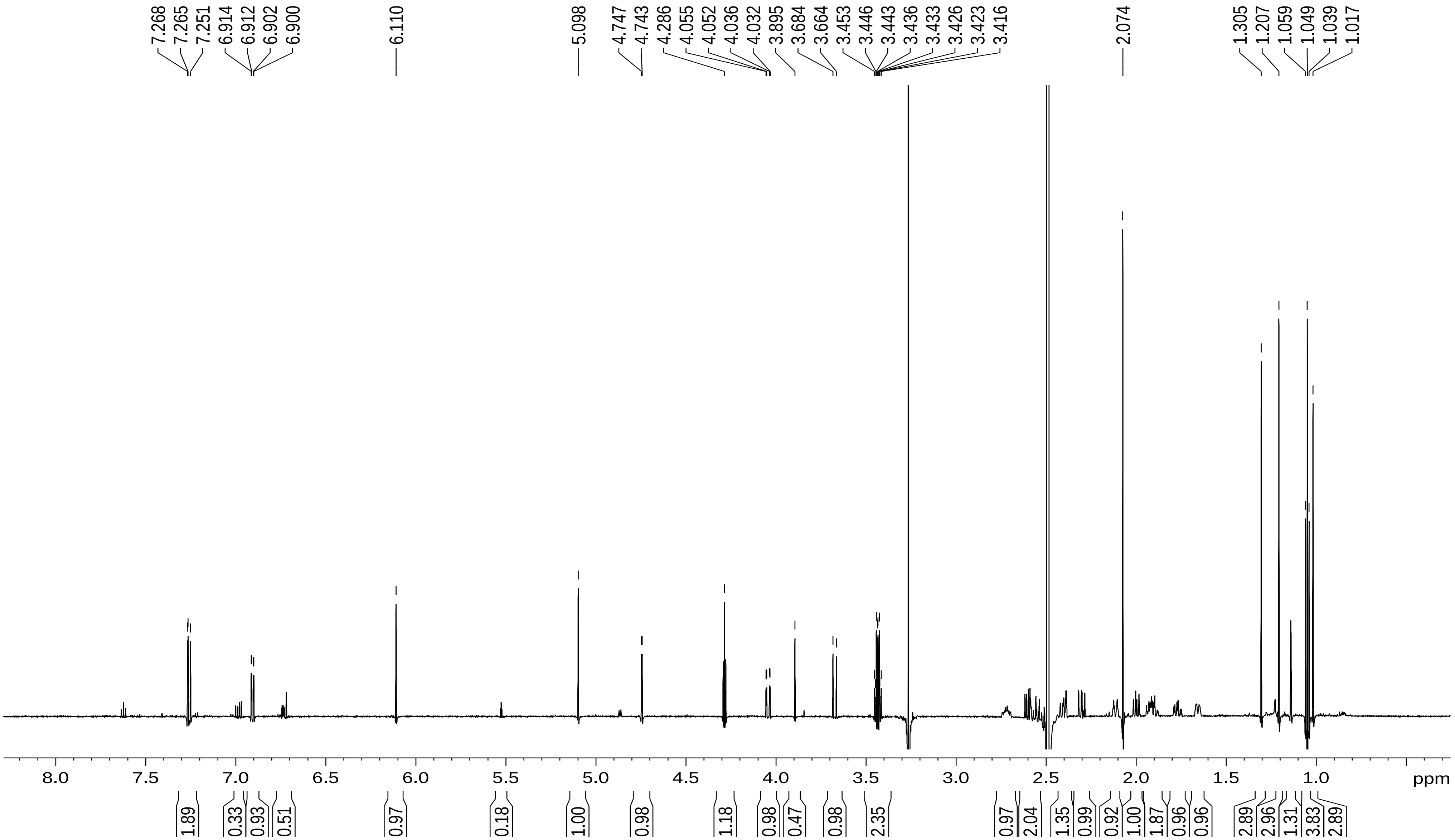

Mar. Drugs 2018, 16, 232; doi:10.3390/md16070232

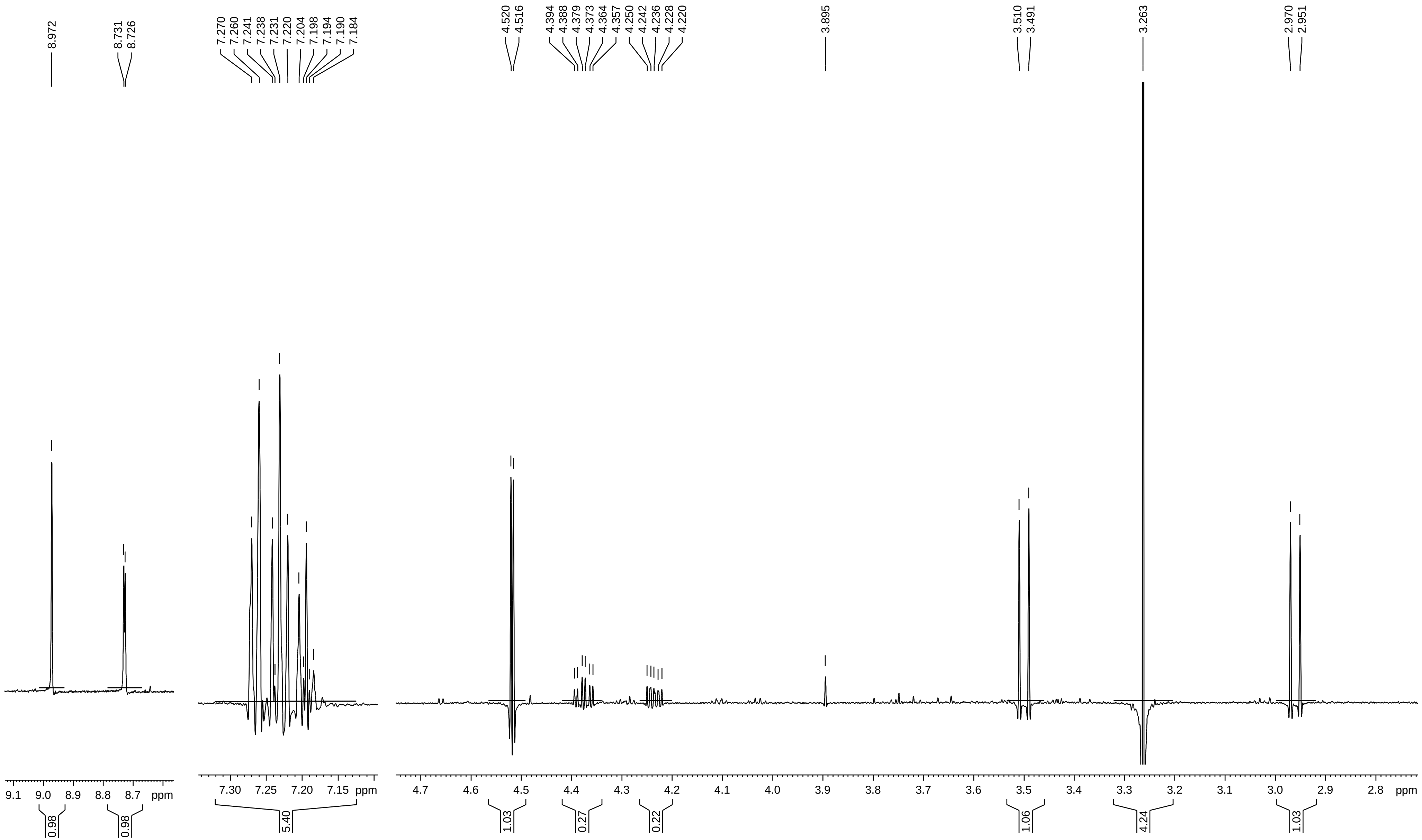
8.972
8.731
8.726
7.270
7.260
7.241
7.238
7.231
7.220
7.204
7.198
7.194
7.190
7.184
4.520
4.516
4.394
4.388
4.379
4.373
4.364
4.357
4.250
4.242
4.236
4.228
4.220
3.895
3.510
3.491
3.263
2.970
2.951
ppm
0.98
0.98
5.40
1.03
0.27
0.22
1.06
4.24
1.03

Figure S2. ^{13}C NMR (125 MHz, DMSO-d_6) spectrum of asperindole A (**1**)

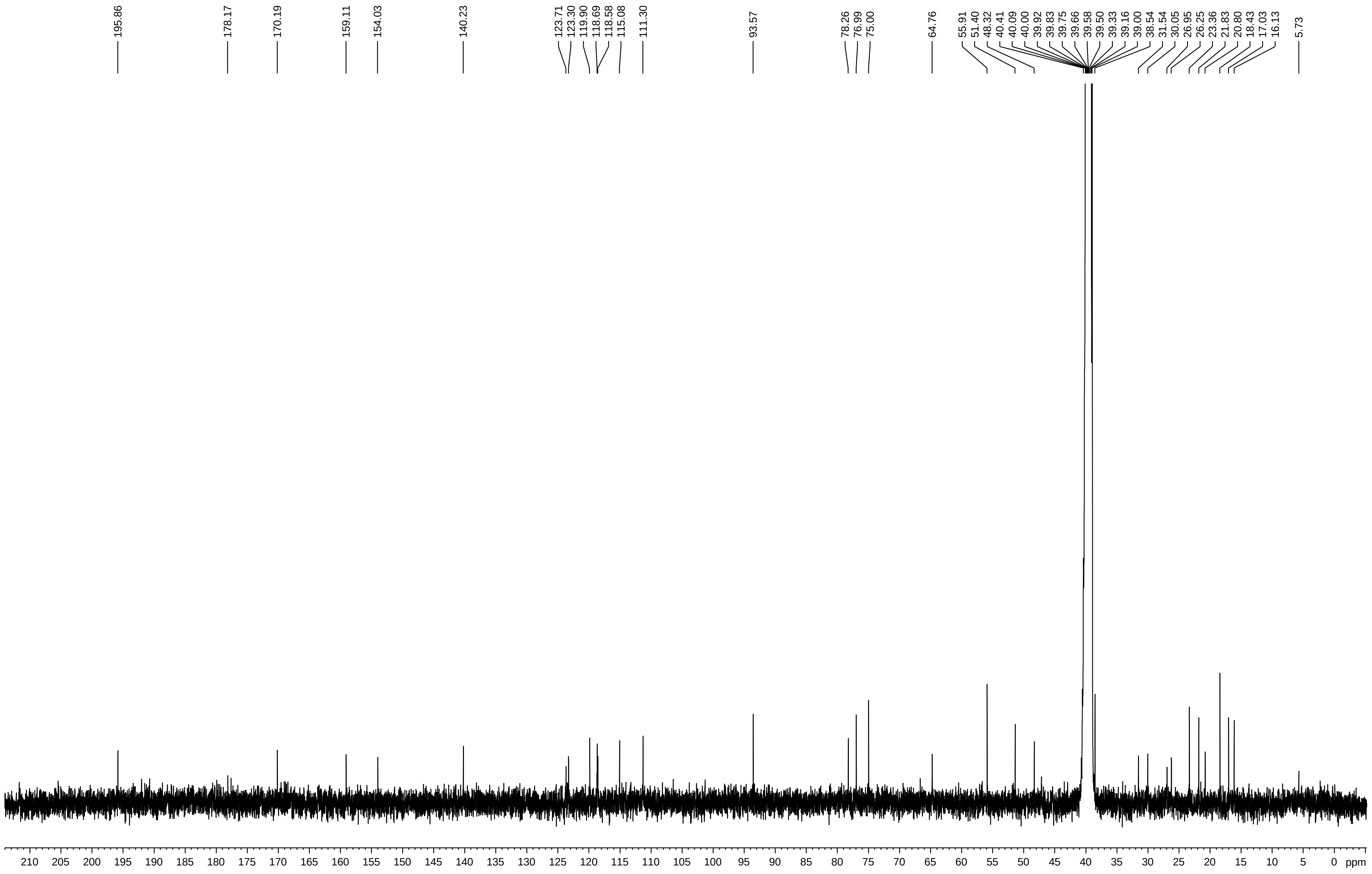

Mar. Drugs 2018, 16, 232; doi:10.3390/md16070232

Figure S3. DEPT-135 (125 MHz, DMSO-d_6) spectrum of asperindole A (**1**)

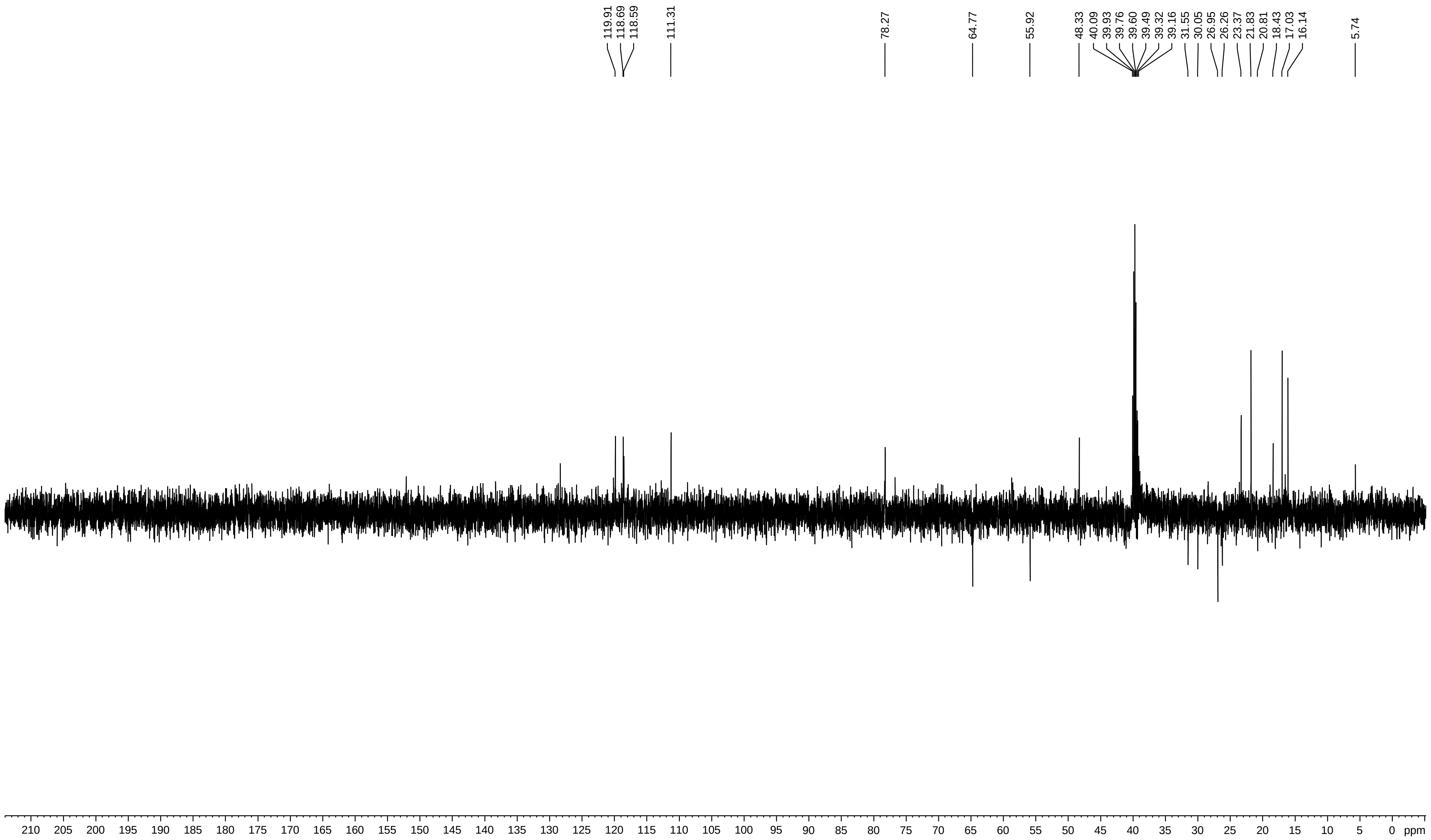

Figure S4. HSQC (700 MHz, DMSO-d_6) spectrum of asperindole A (**1**)

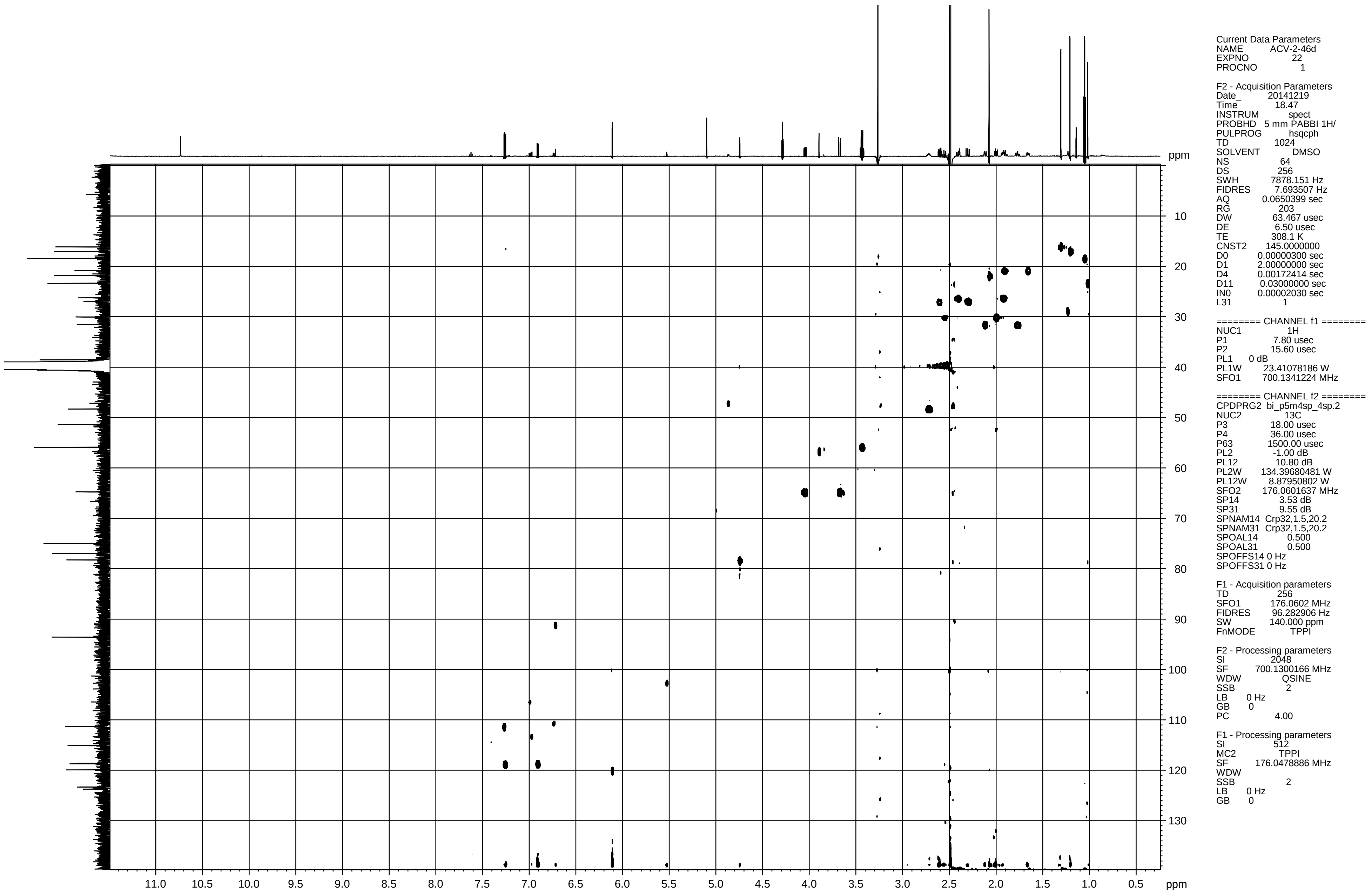

Figure S5. HMBC (700 MHz, DMSO-d_6) spectrum of asperindole A (**1**)

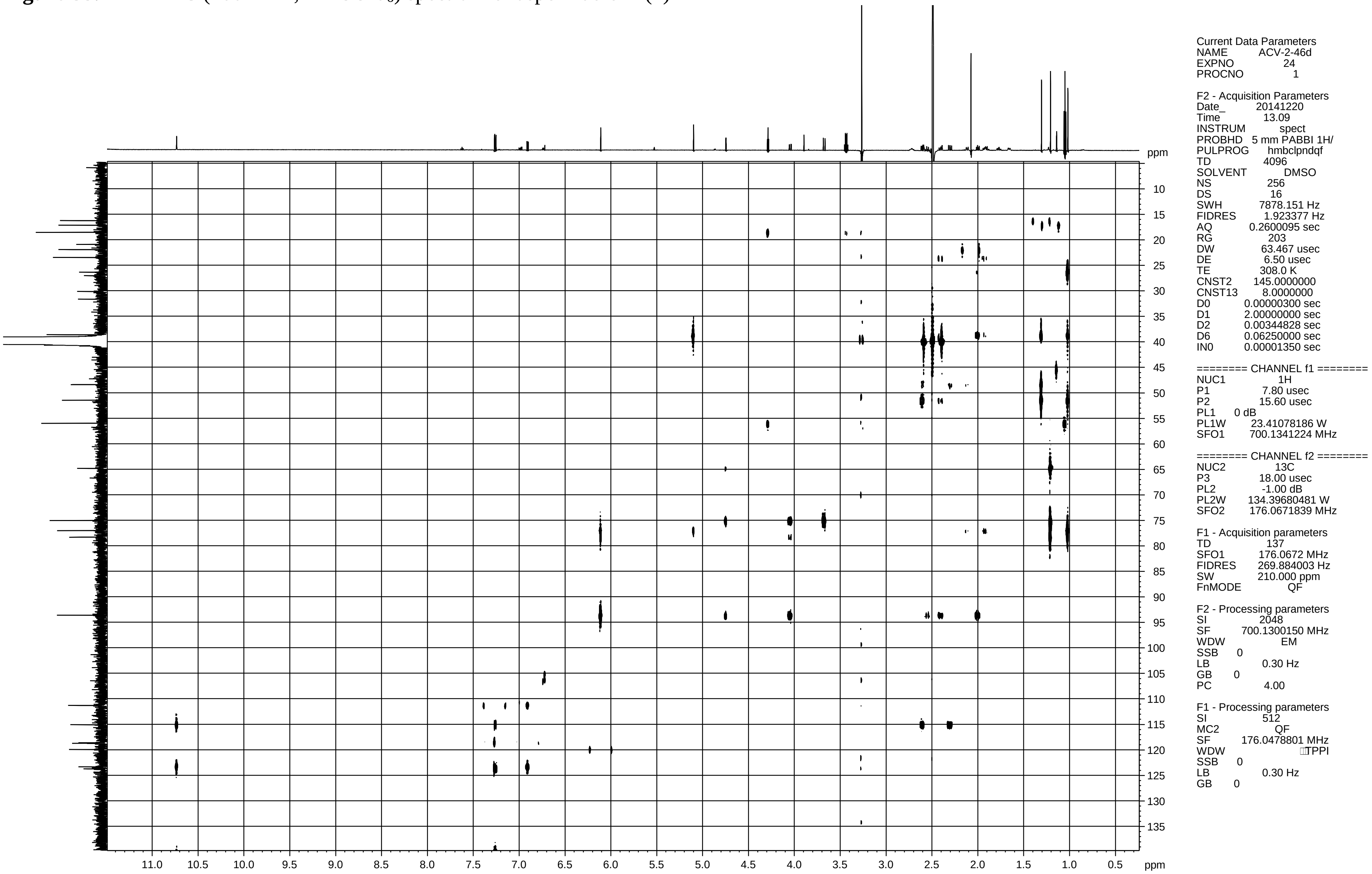

Figure S6. ROESY (700 MHz, DMSO-d_6) spectrum of asperindole A (**1**)

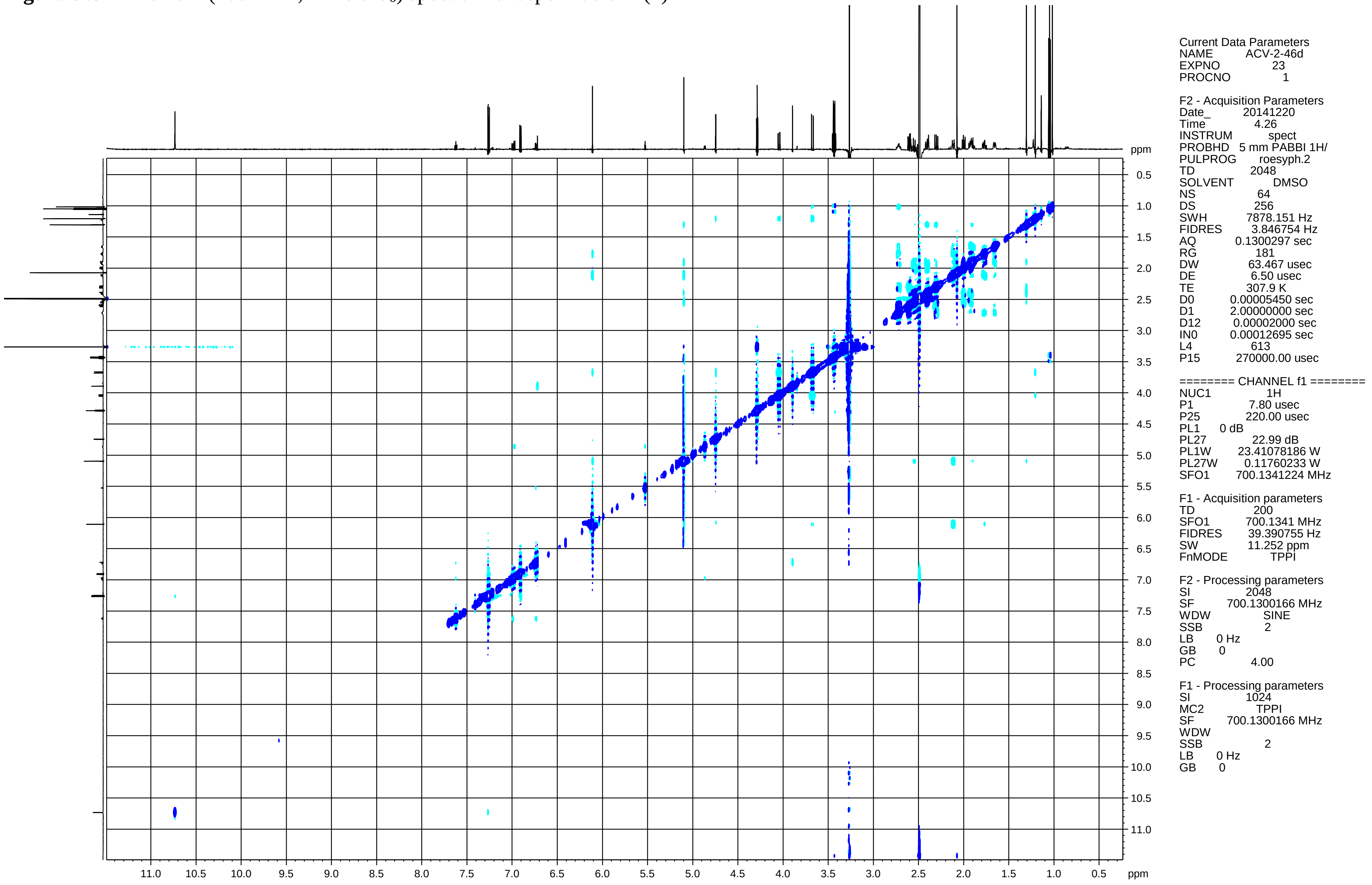

Figure S7. ^{1}H NMR (500 MHz, DMSO-d$_6$) spectrum of asperindole B (**2**)

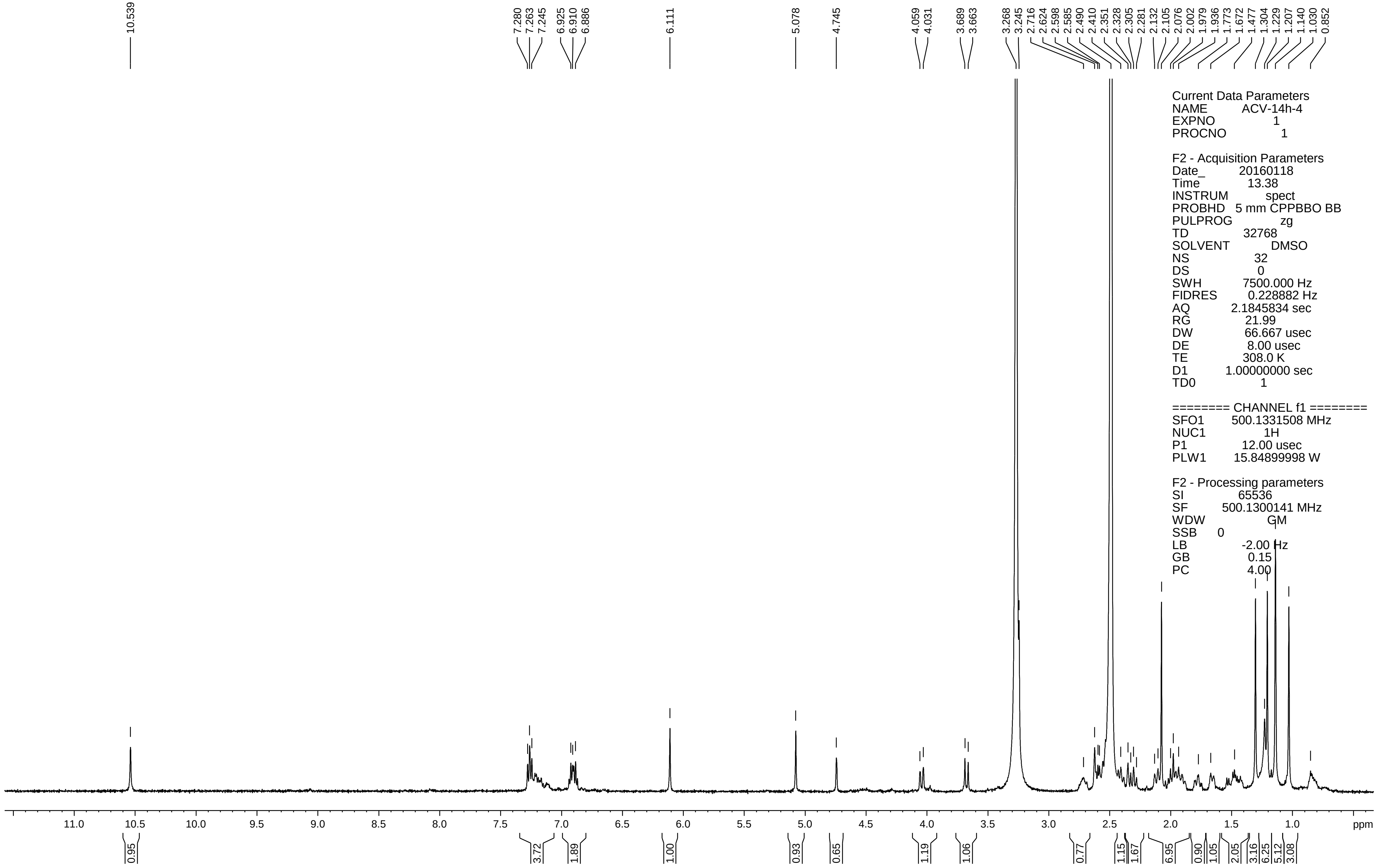

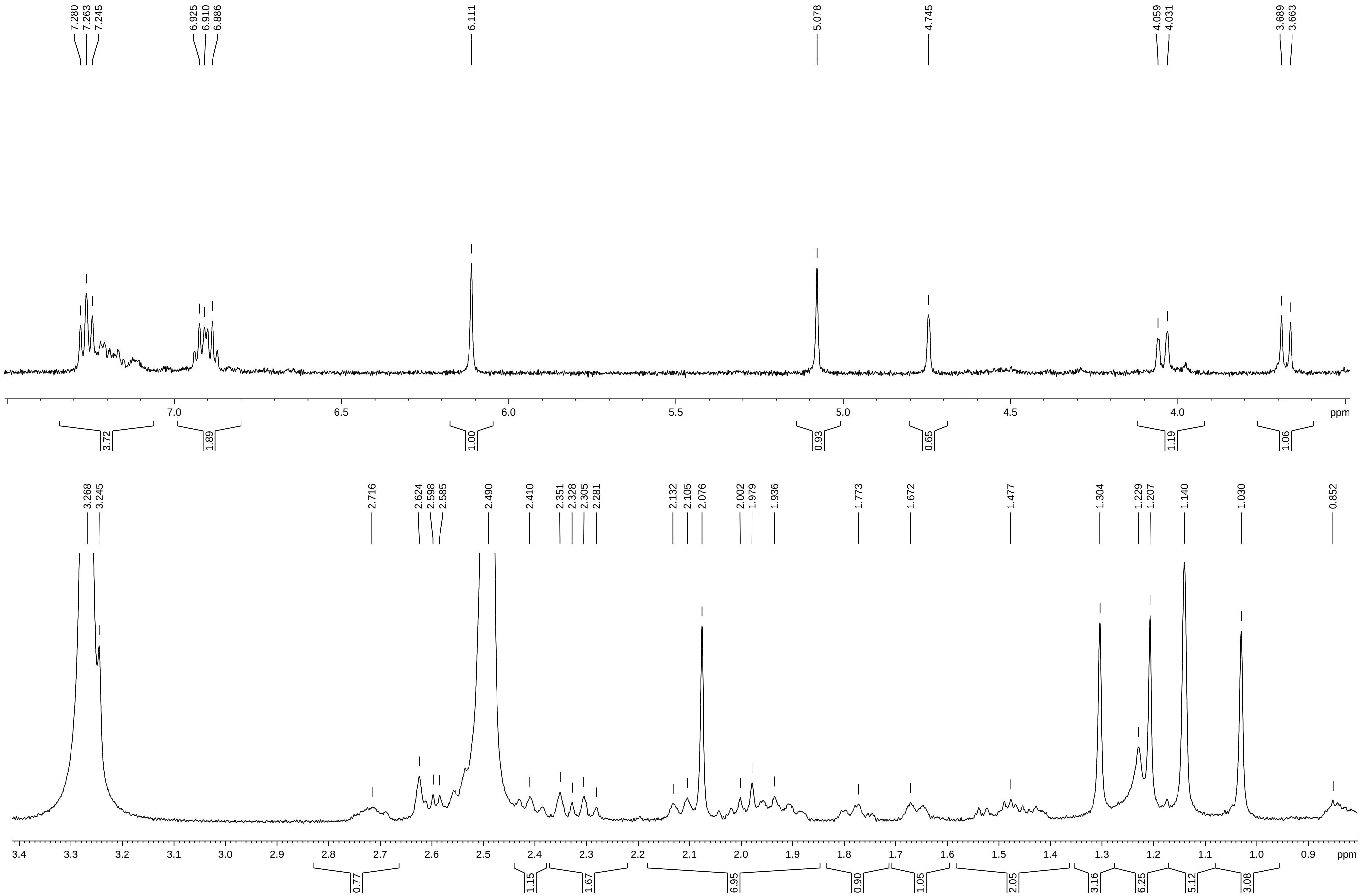
7.280 7.263 7.245 6.925 6.910 6.886 6.111 5.078 4.745 4.059 4.031 3.689 3.663
7.0 6.5 6.0 5.5 5.0 4.5 4.0 ppm
3.72 1.89 1.00 0.93 0.65 1.19 1.06
3.268 3.245 2.716 2.624 2.598 2.585 2.490 2.410 2.351 2.328 2.305 2.281 2.132 2.105 2.076 2.002 1.979 1.936 1.773 1.672 1.477 1.304 1.229 1.207 1.140 1.030 0.852
3.4 3.3 3.2 3.1 3.0 2.9 2.8 2.7 2.6 2.5 2.4 2.3 2.2 2.1 2.0 1.9 1.8 1.7 1.6 1.5 1.4 1.3 1.2 1.1 1.0 0.9 ppm
0.77 1.15 1.67 6.95 0.90 1.05 2.05 3.16 6.25 5.12 3.08

Figure S8. ^{13}C NMR (125 MHz, DMSO-d_6) spectra of asperindole B (**2**)

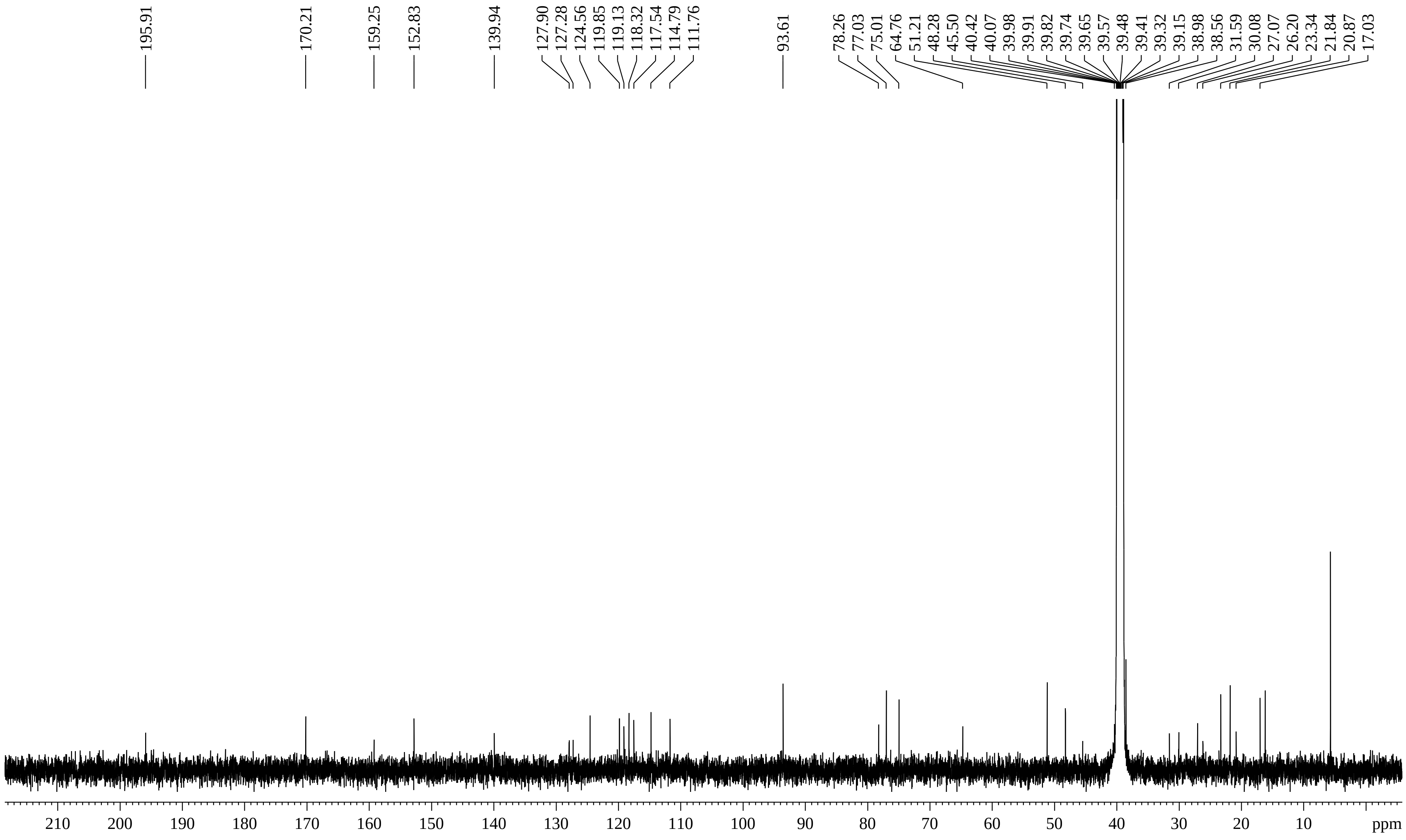

Mar. Drugs 2018, 16, 232; doi:10.3390/md16070232

Figure S9. DEPT-135 (125 MHz, DMSO-d_6) spectrum of asperindole B (**2**)

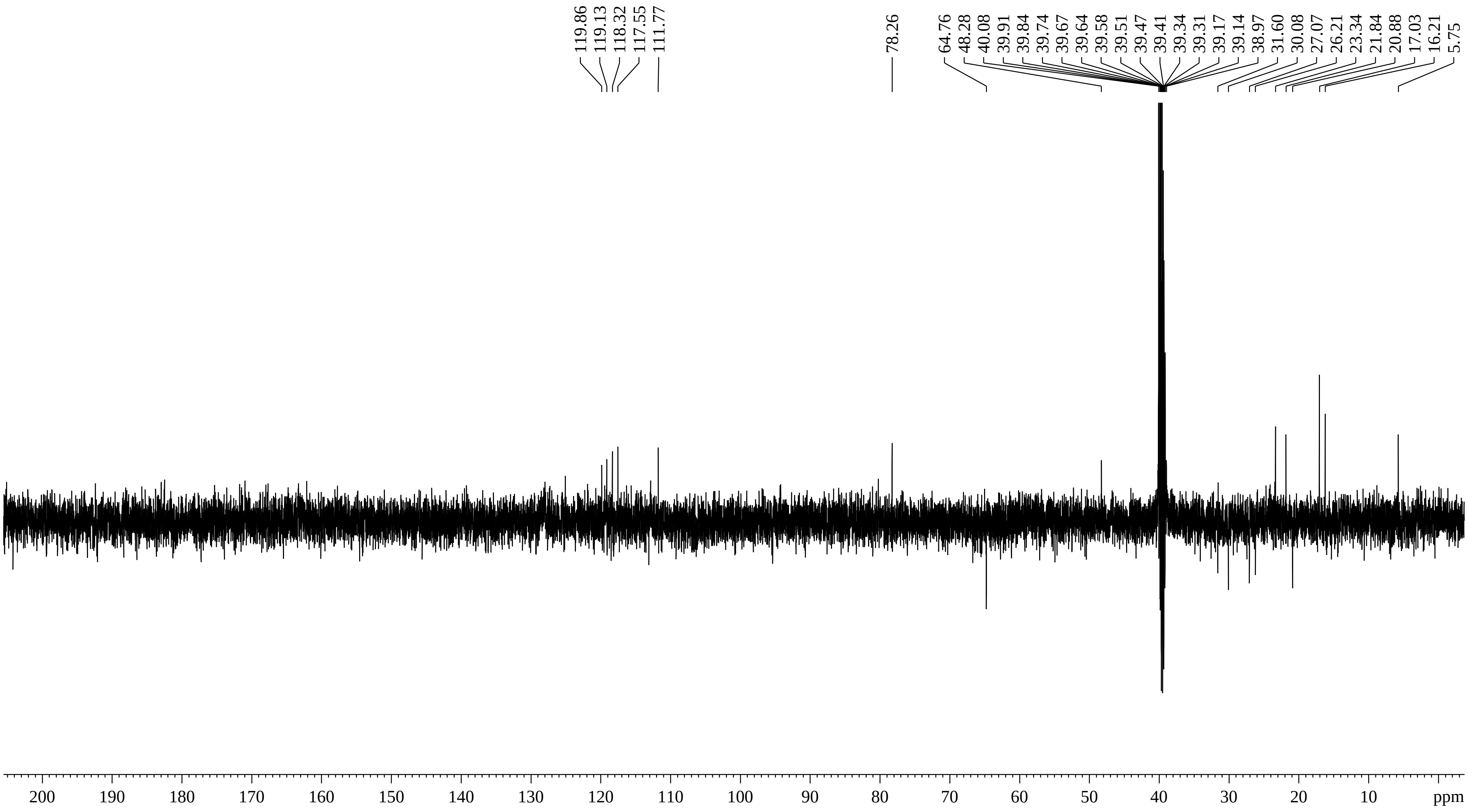

Figure S10. ^{1}H-^{1}H COSY (500 MHz, DMSO-d_6) spectrum of asperindole B (**2**)

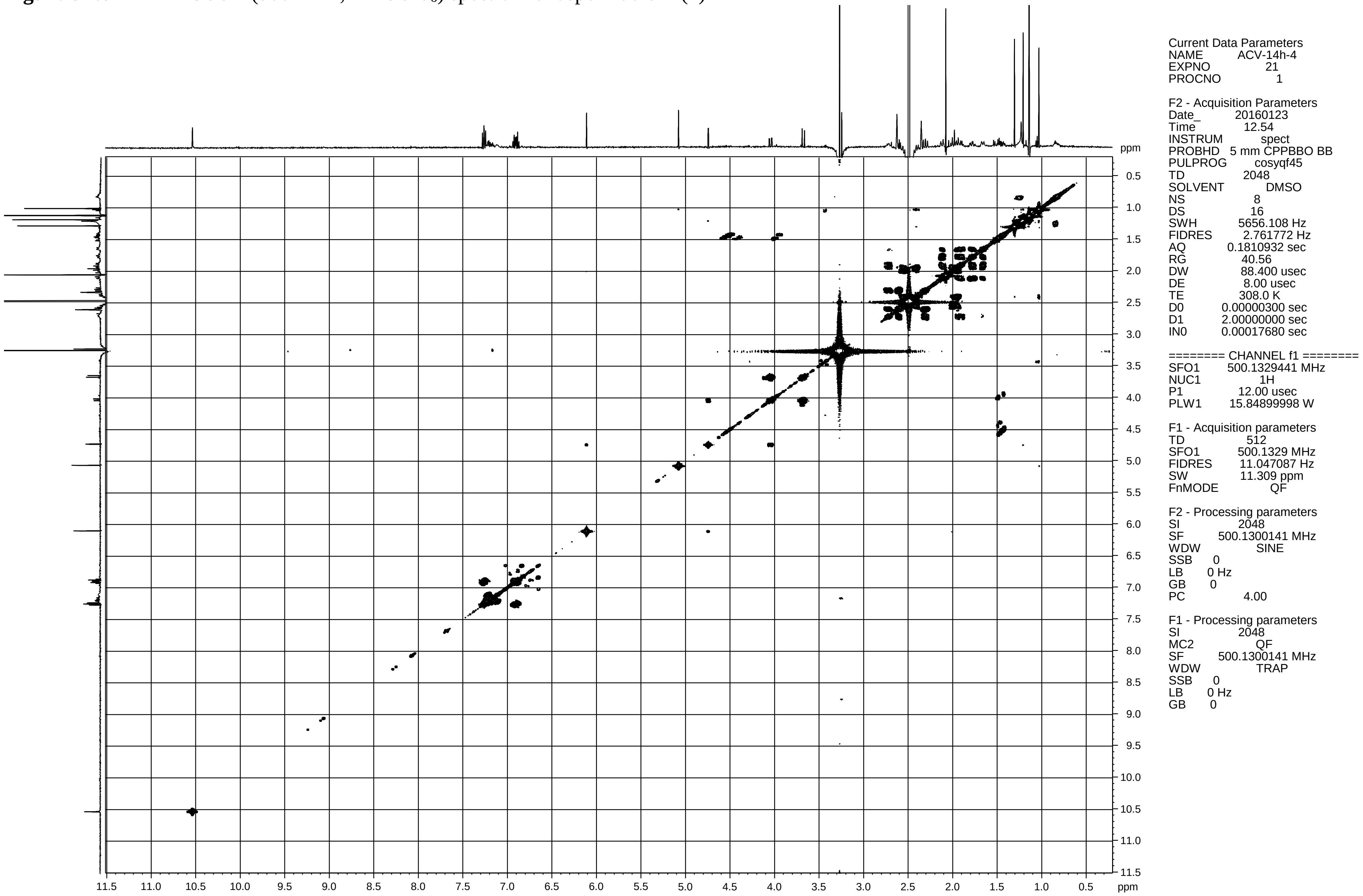

Figure S11. HSQC (500 MHz, DMSO-d_6) spectrum of asperindole B (**2**)

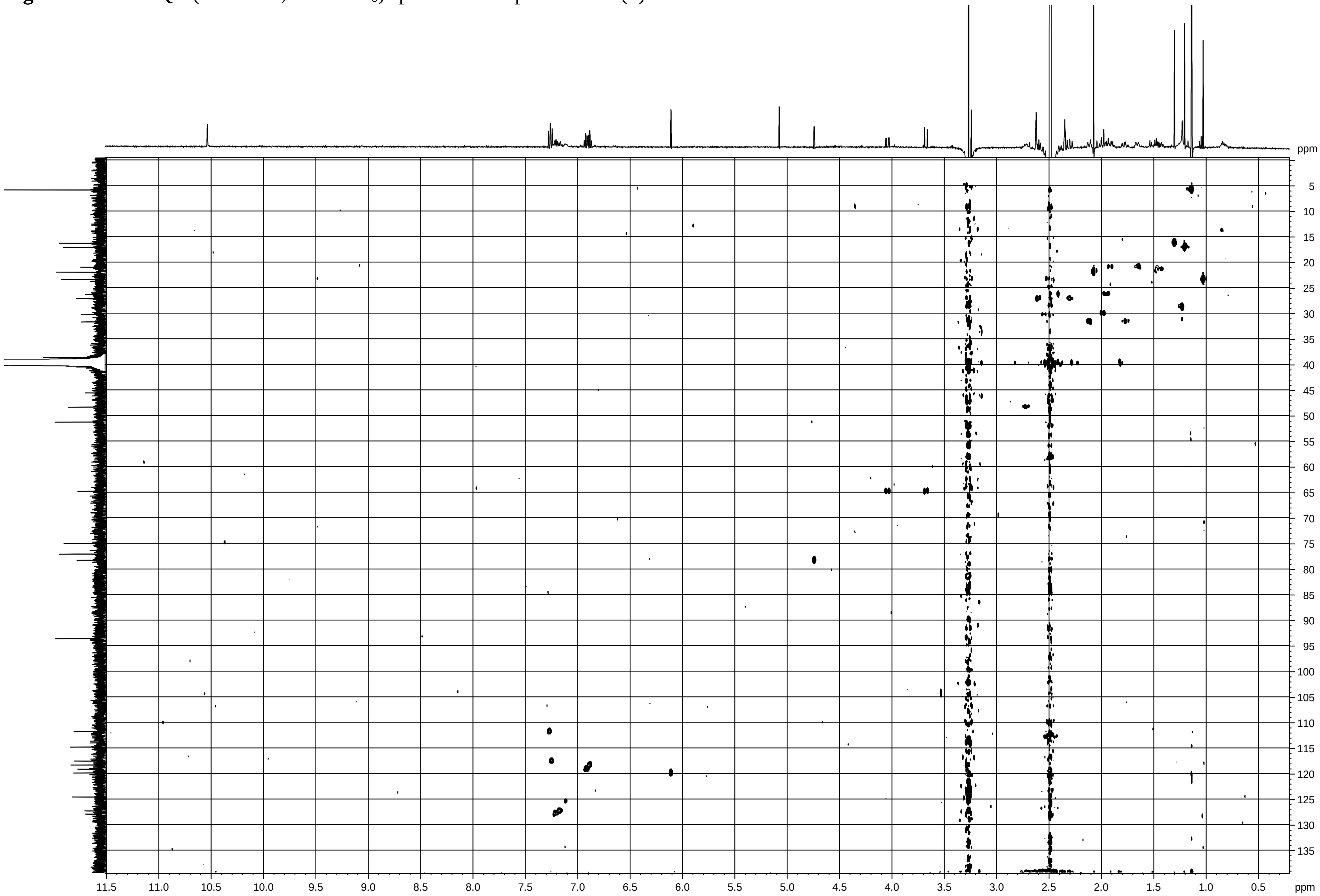

Mar. Drugs 2018, 16, 232; doi:10.3390/md16070232

Figure S12. HMBC (500 MHz, DMSO-d_6) spectrum of asperindole B (**2**)

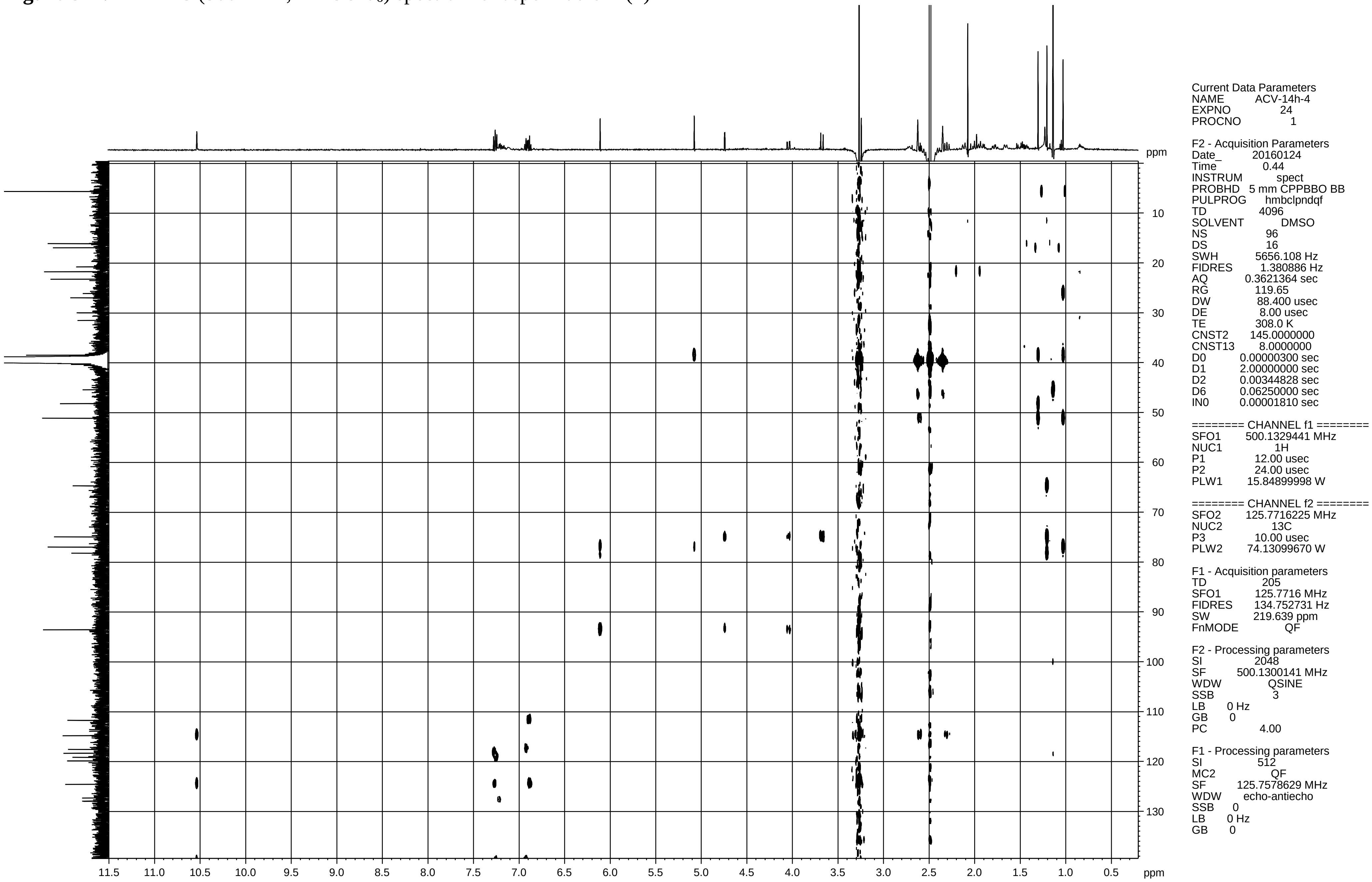

Figure S13. ROESY (500 MHz, DMSO-d_6) spectrum of asperindole B (**2**)

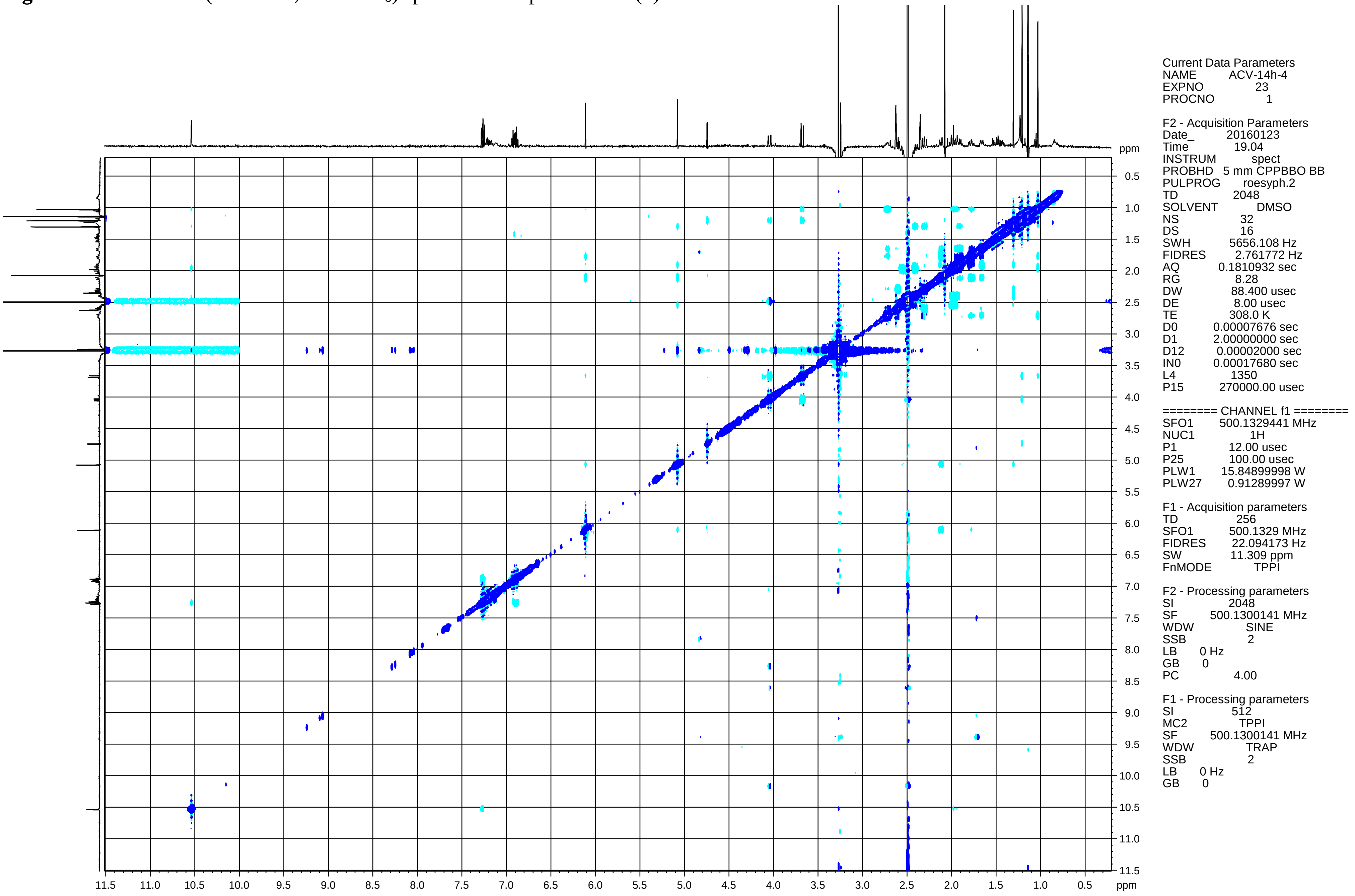

Figure S14. ^{1}H NMR (500 MHz, DMSO-d6) spectrum of asperindole C (**3**)

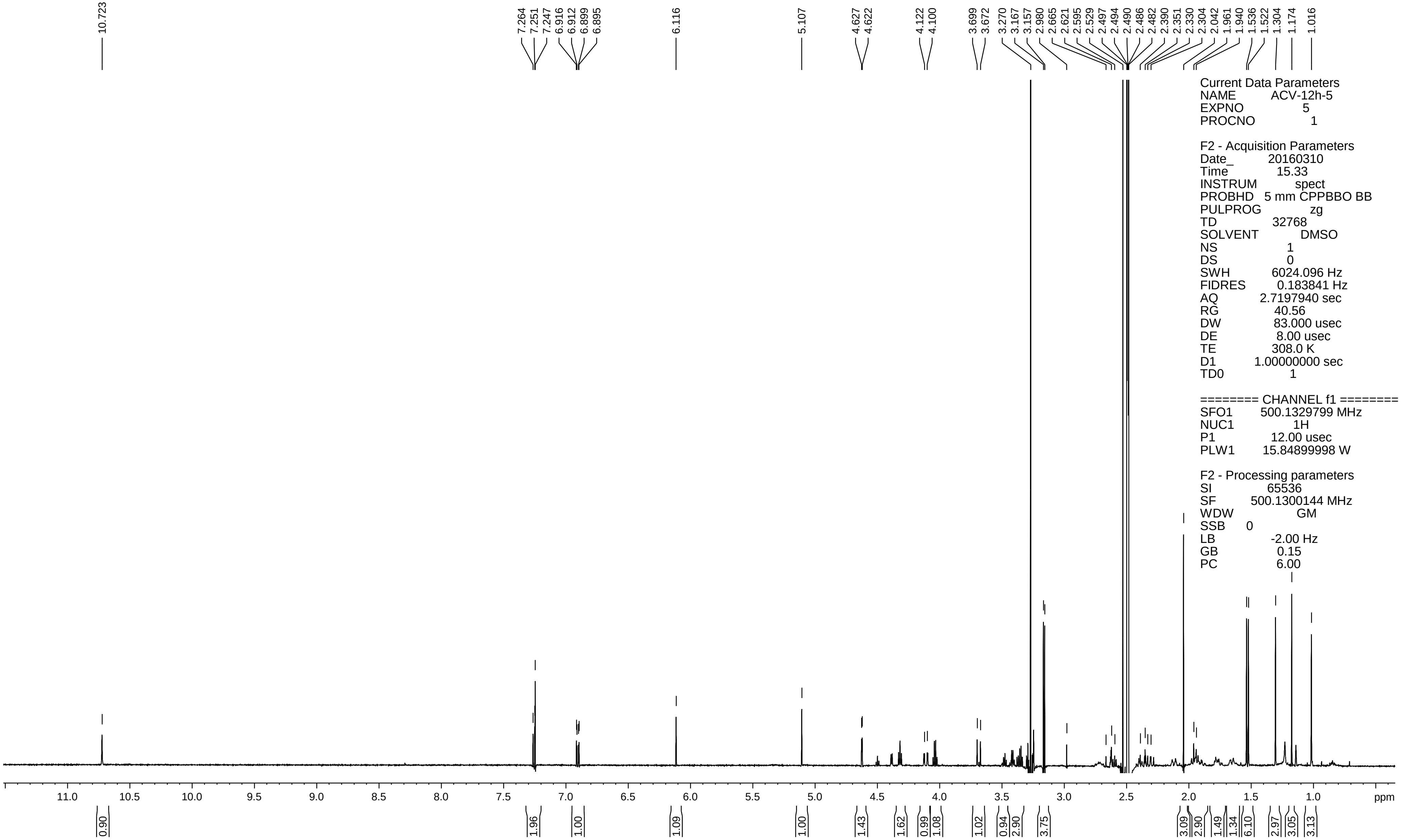

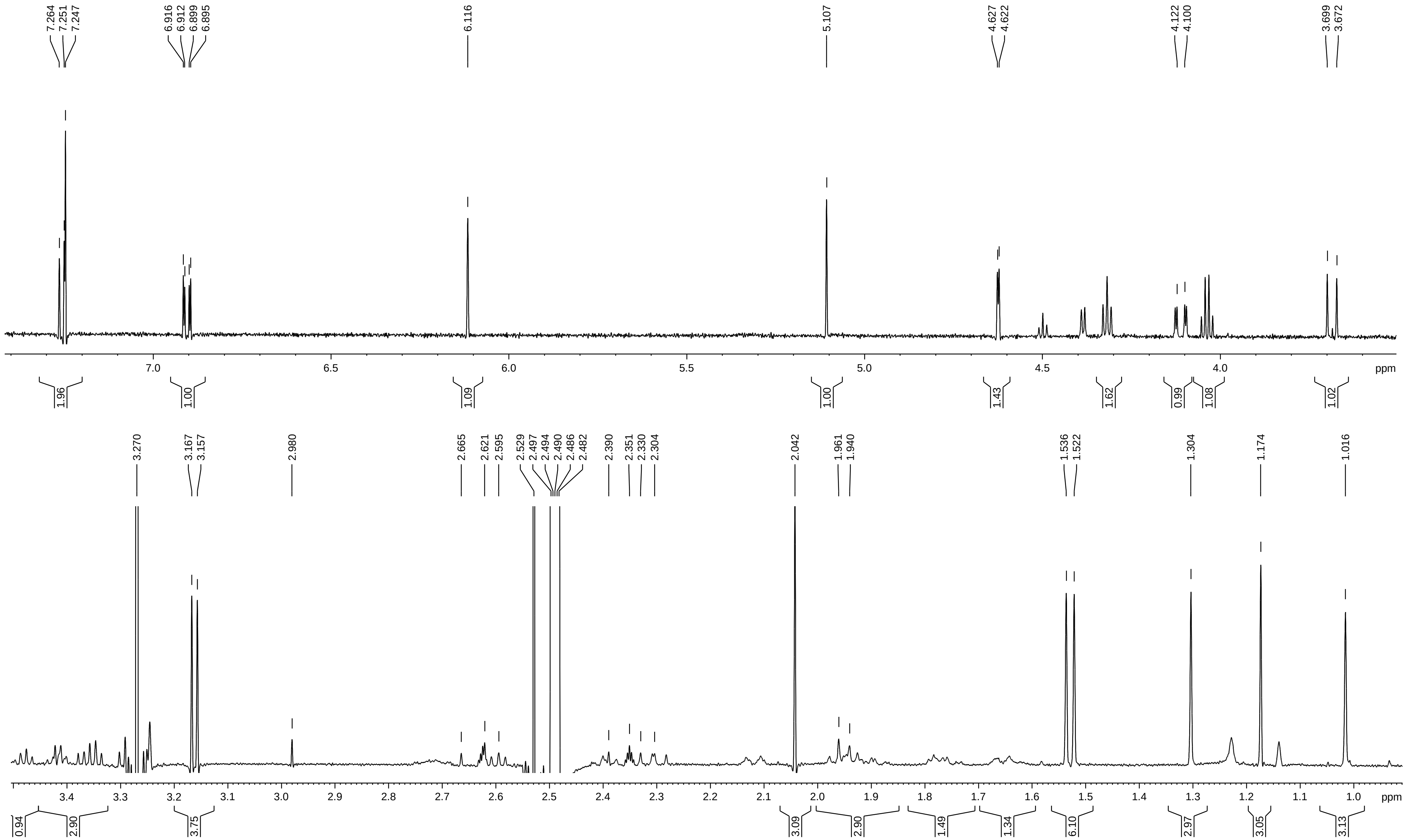
7.264
7.251
7.247
6.916
6.912
6.899
6.895
6.116
5.107
4.627
4.622
4.122
4.100
3.699
3.672
7.0
6.5
6.0
5.5
5.0
4.5
4.0
ppm
1.96
1.00
1.09
1.00
1.43
1.62
0.99
1.08
1.02
3.270
3.167
3.157
2.980
2.665
2.621
2.595
2.529
2.497
2.494
2.490
2.486
2.482
2.390
2.351
2.330
2.304
2.042
1.961
1.940
1.536
1.522
1.304
1.174
1.016
3.4
3.3
3.2
3.1
3.0
2.9
2.8
2.7
2.6
2.5
2.4
2.3
2.2
2.1
2.0
1.9
1.8
1.7
1.6
1.5
1.4
1.3
1.2
1.1
1.0
ppm
0.94
2.90
3.75
3.09
2.90
1.49
1.34
6.10
2.97
3.05
3.13

Figure S15. ^{13}C NMR (125 MHz, DMSO-d6) spectra of asperindole C (**3**)

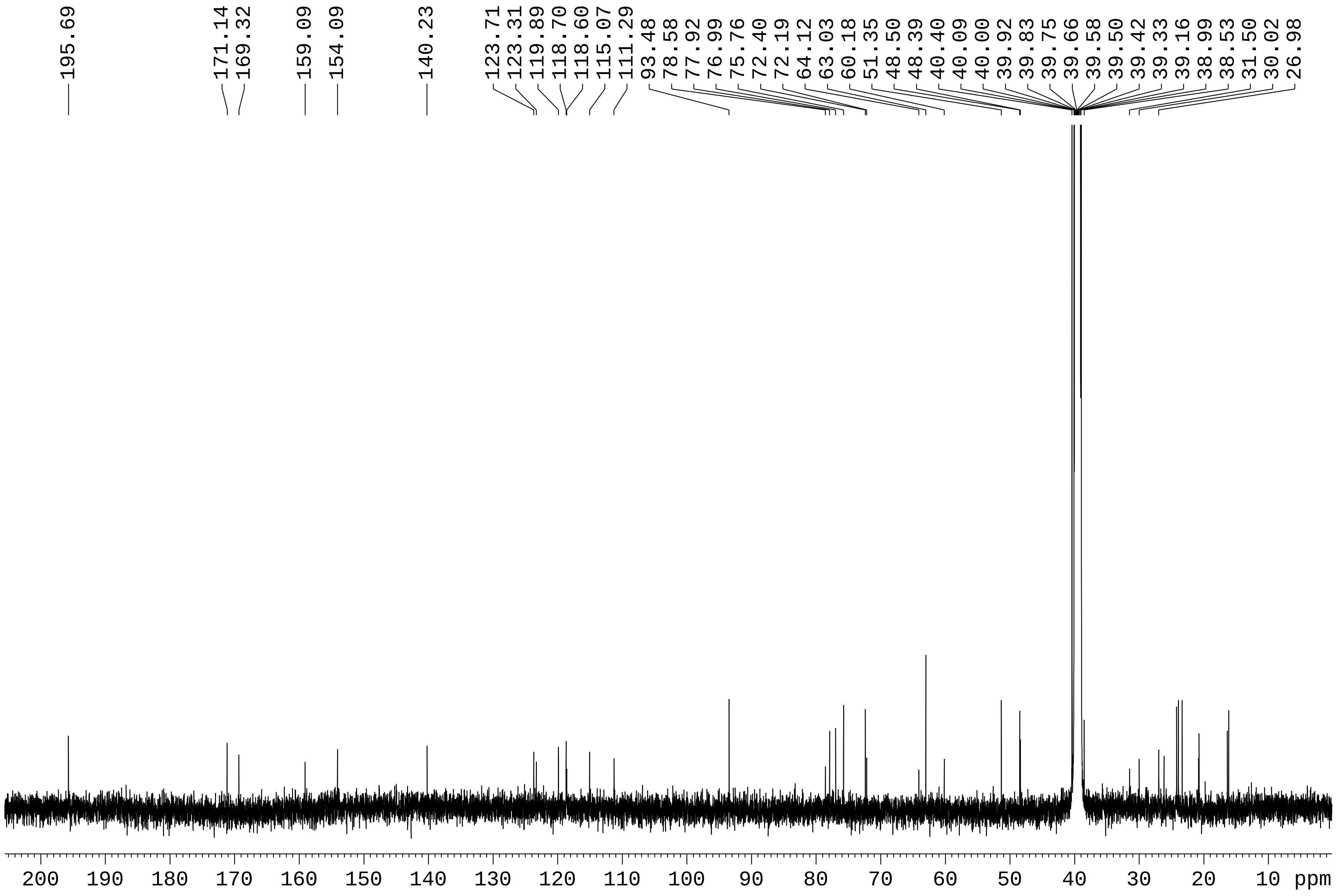

Mar. Drugs 2018, 16, 232; doi:10.3390/md16070232

Figure S16. DEPT-135 (125 MHz, DMSO-d_6) spectrum of asperindole C (**3**)

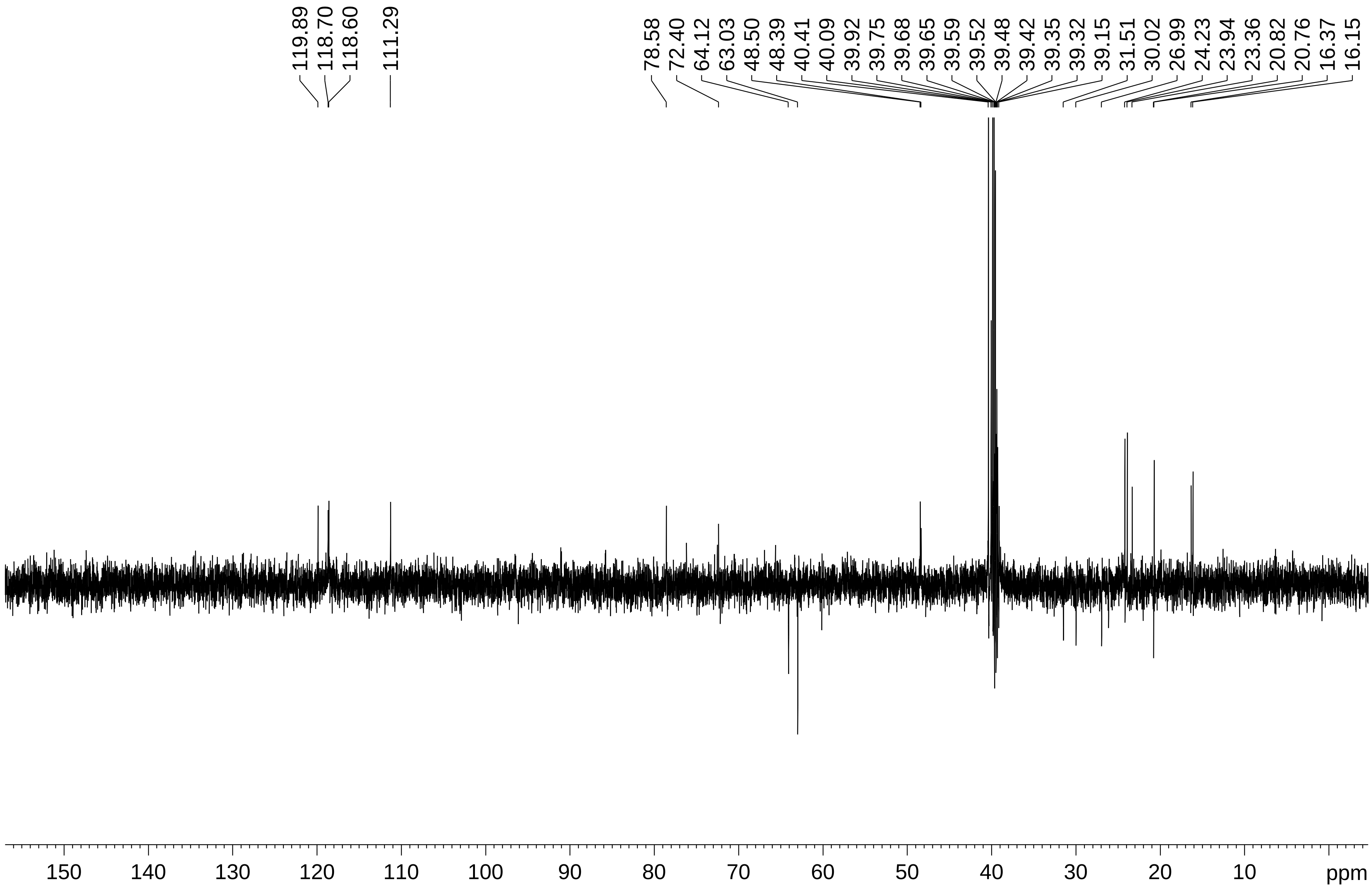

Mar. Drugs 2018, 16, 232; doi:10.3390/md16070232

Figure S17. ^{1}H-^{1}H COSY (500 MHz, DMSO-d6) spectrum of asperindole C (**3**)

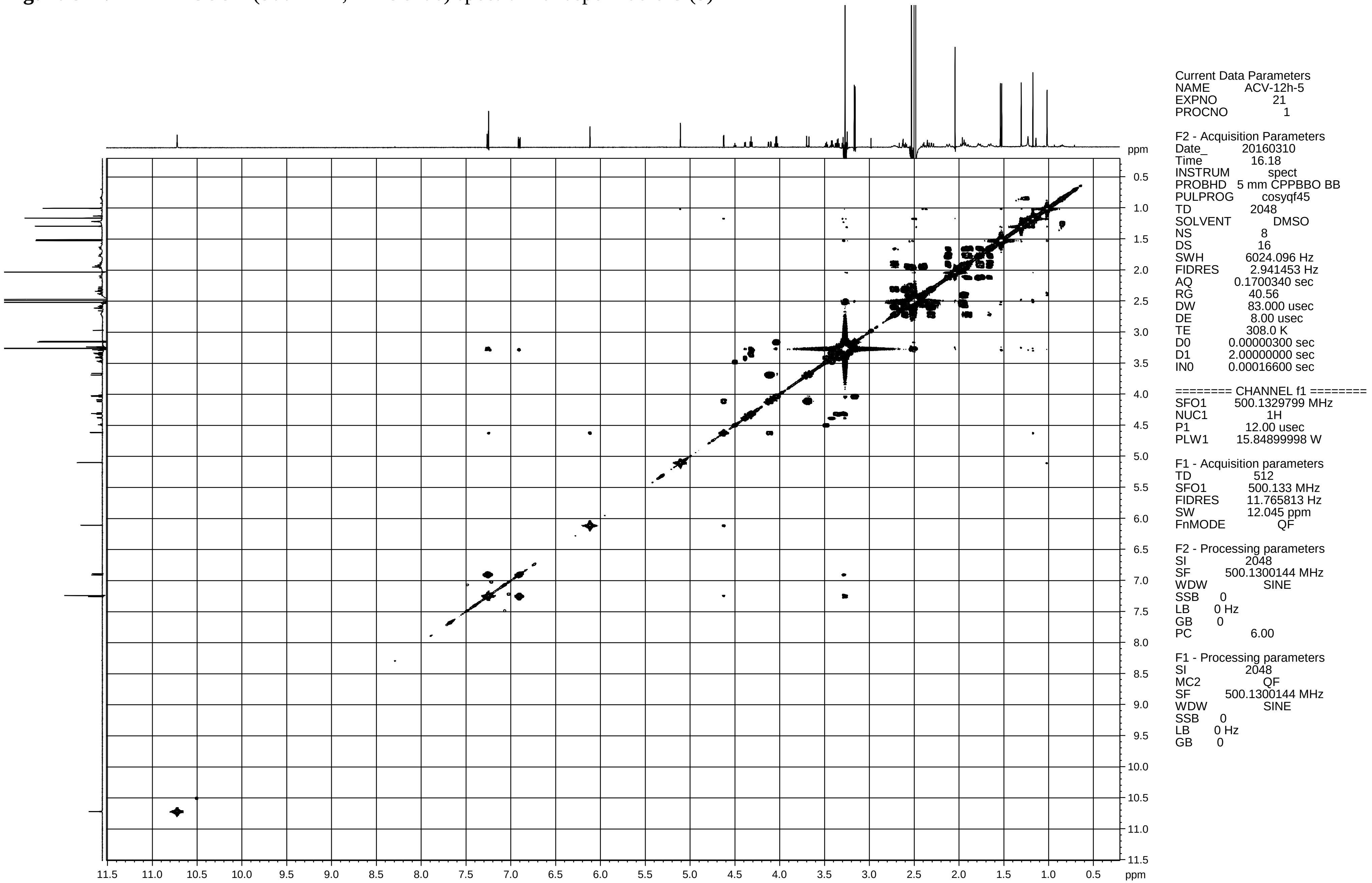

Figure S18. HSQC (500 MHz, DMSO-d6) spectrum of asperindole C (**3**)

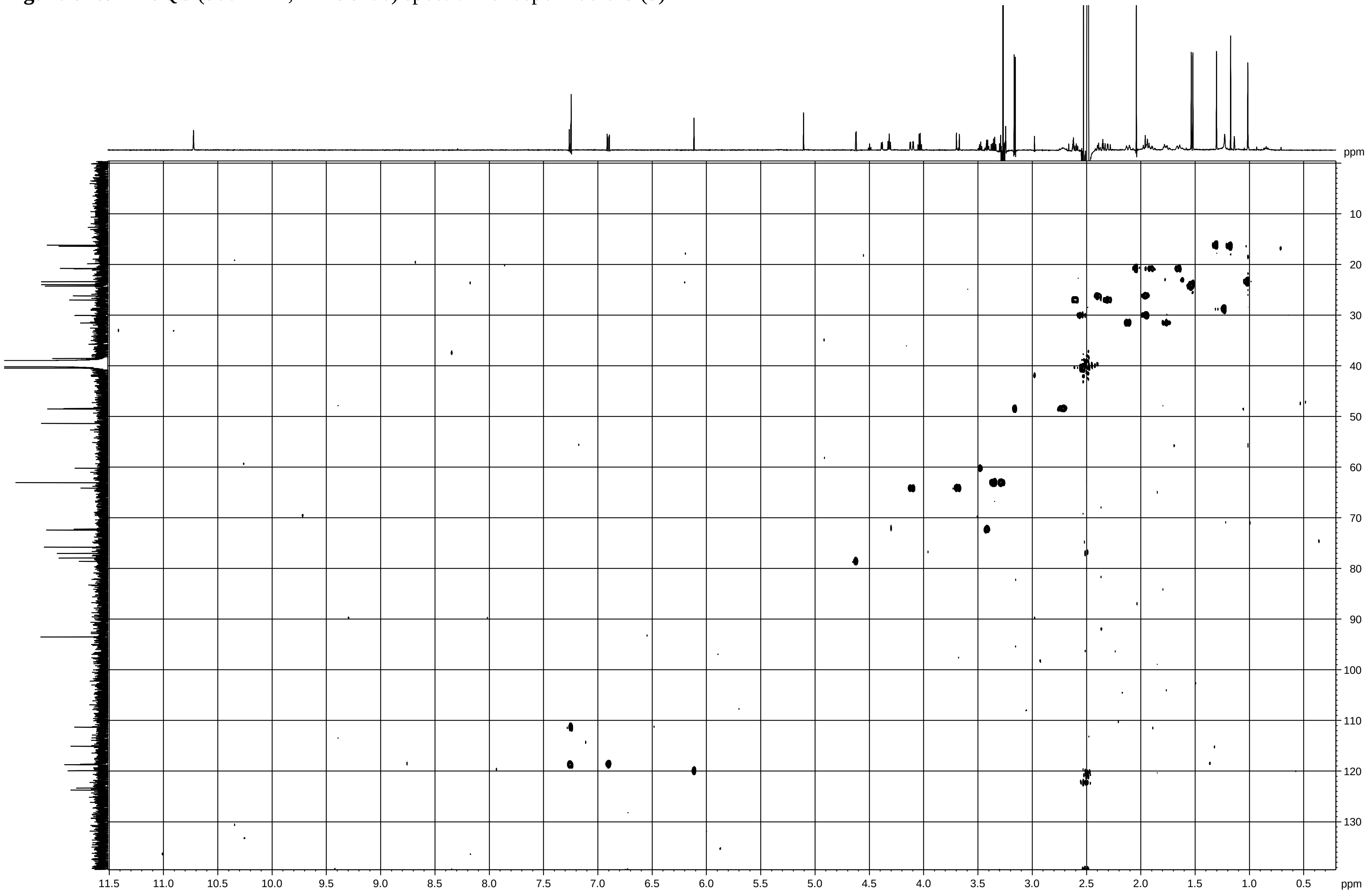

Mar. Drugs 2018, 16, 232; doi:10.3390/md16070232

Figure S19. HMBC (500 MHz, DMSO-d_6) spectrum of asperindole C (**3**)

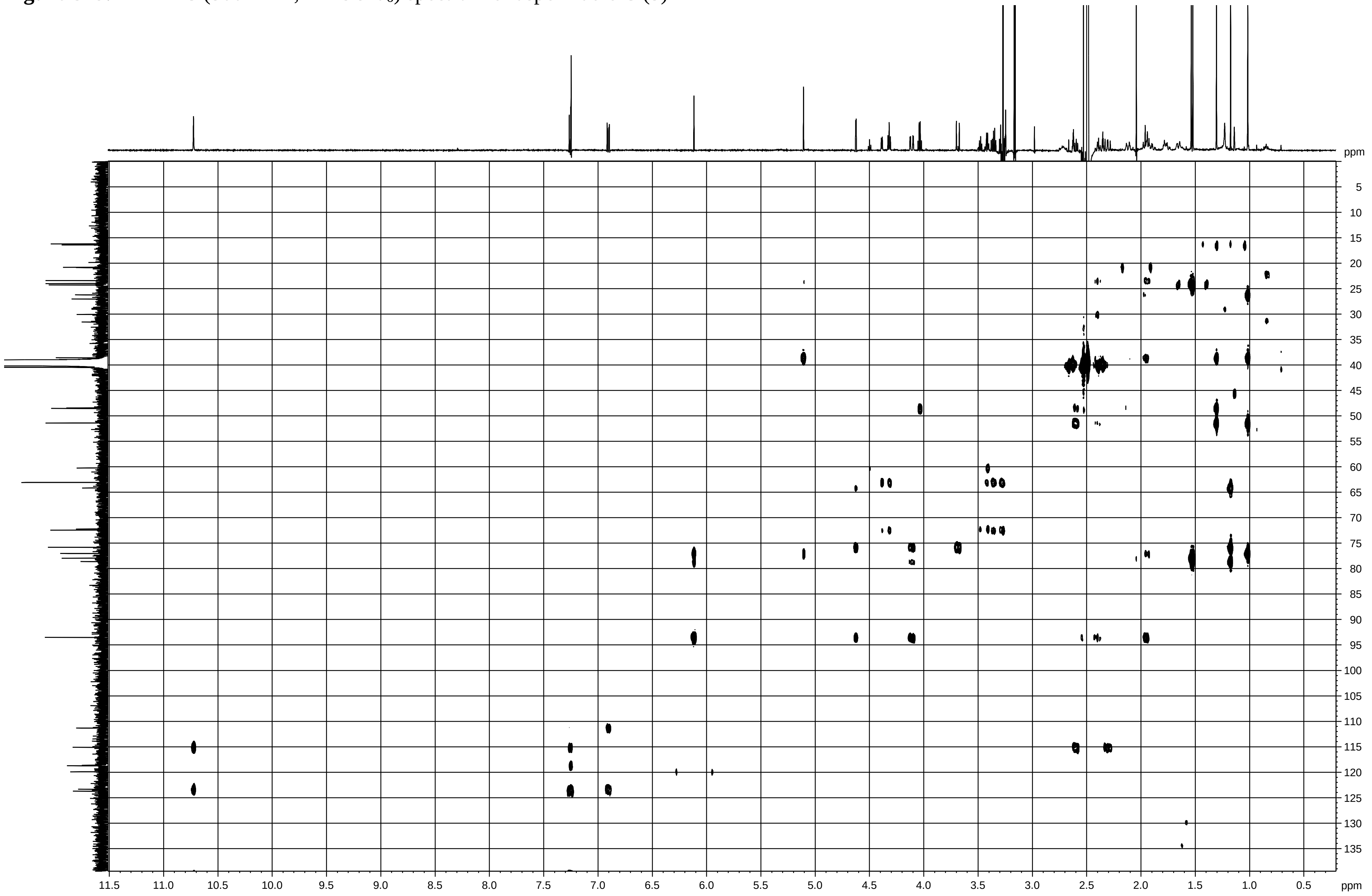

Mar. Drugs 2018, 16, 232; doi:10.3390/md16070232

Figure S20. ROESY (500 MHz, DMSO-d_6) spectrum of asperindole C (**3**)

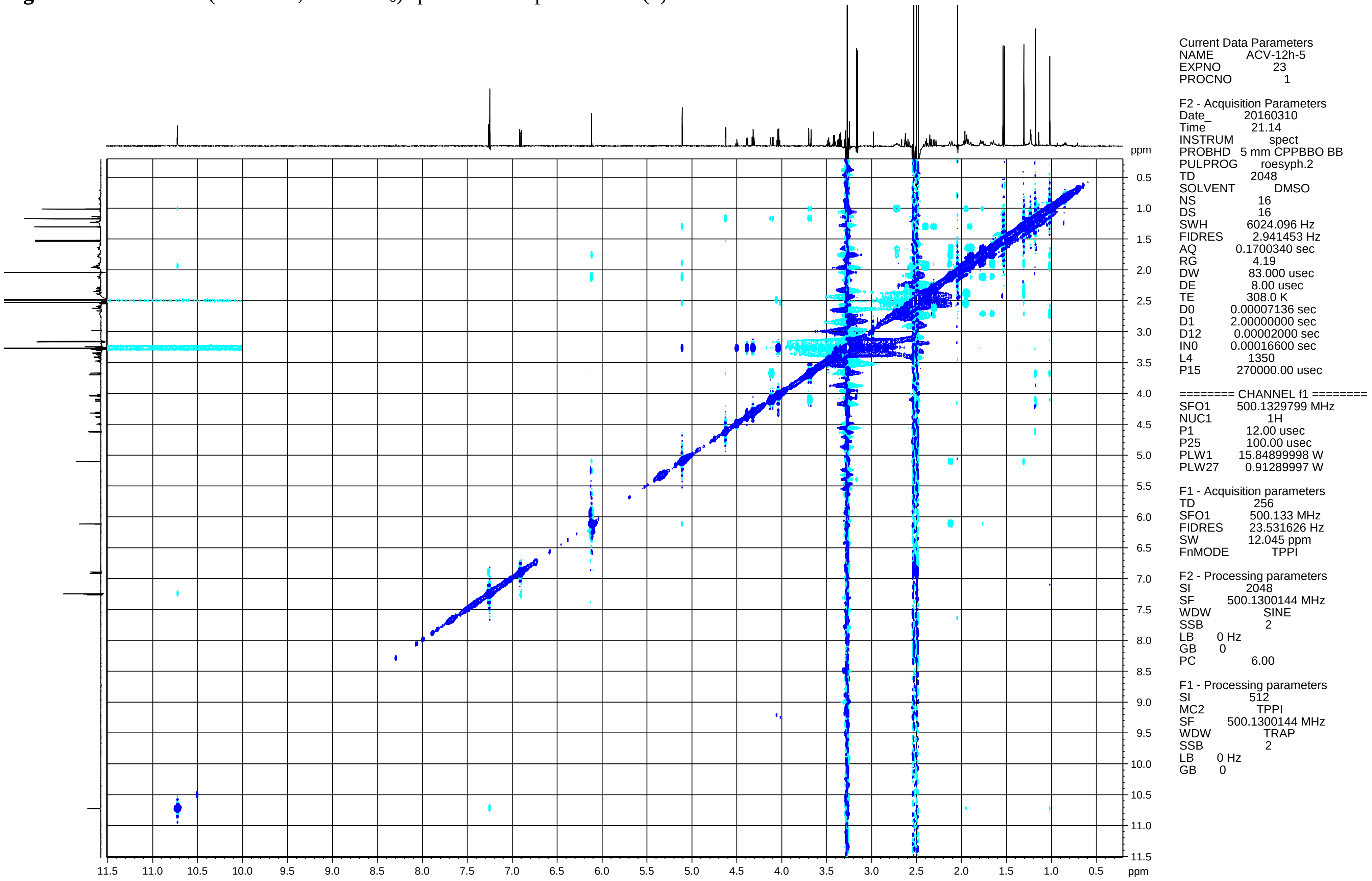

Figure S21. ^{1}H NMR (500 MHz, DMSO-d_6) spectrum of asperindole D (**4**)

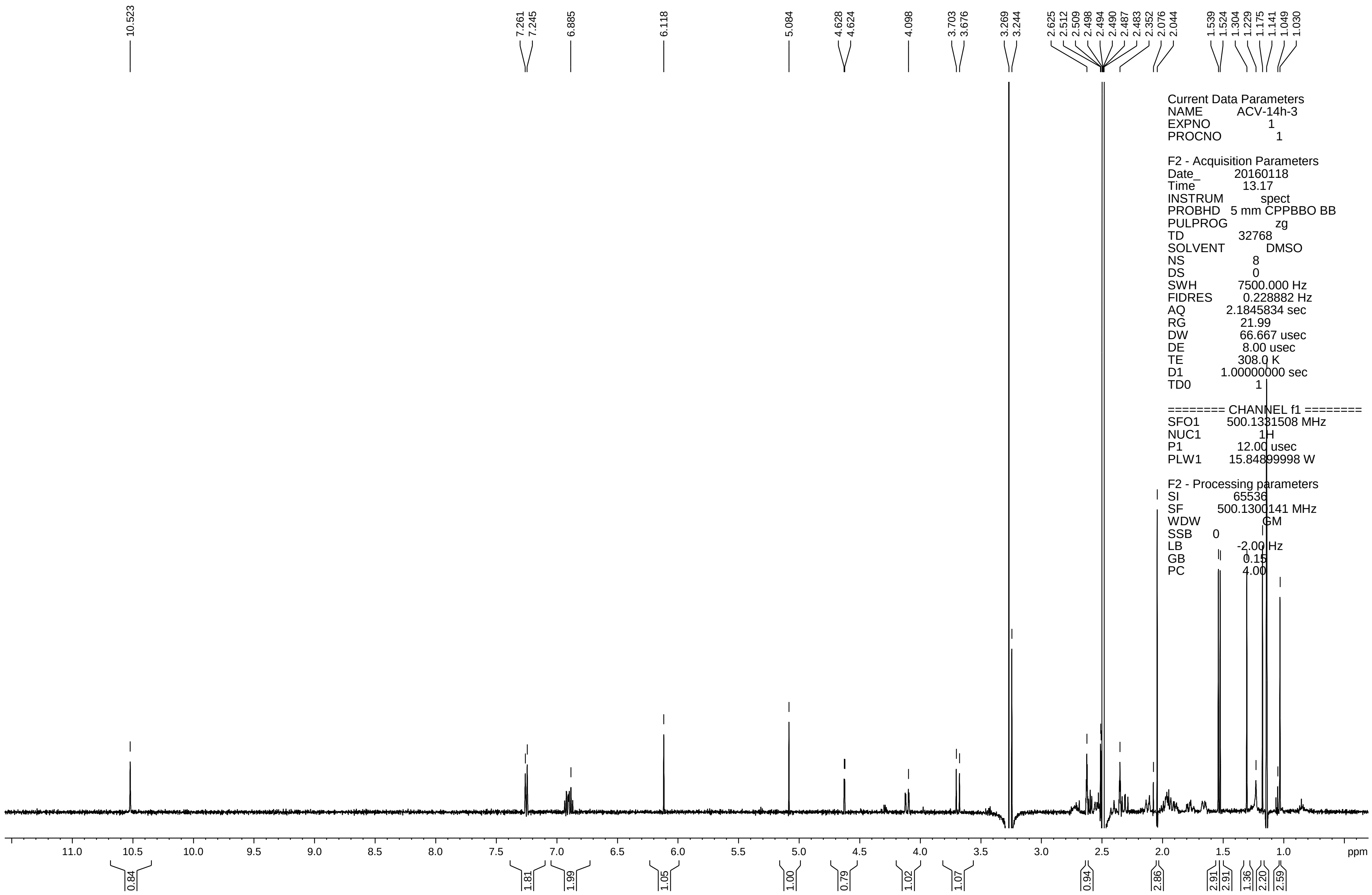

Mar. Drugs 2018, 16, 232; doi:10.3390/md16070232

Figure S22. ^{13}C NMR (125 MHz, DMSO-d_6) spectra of asperindole D (4)

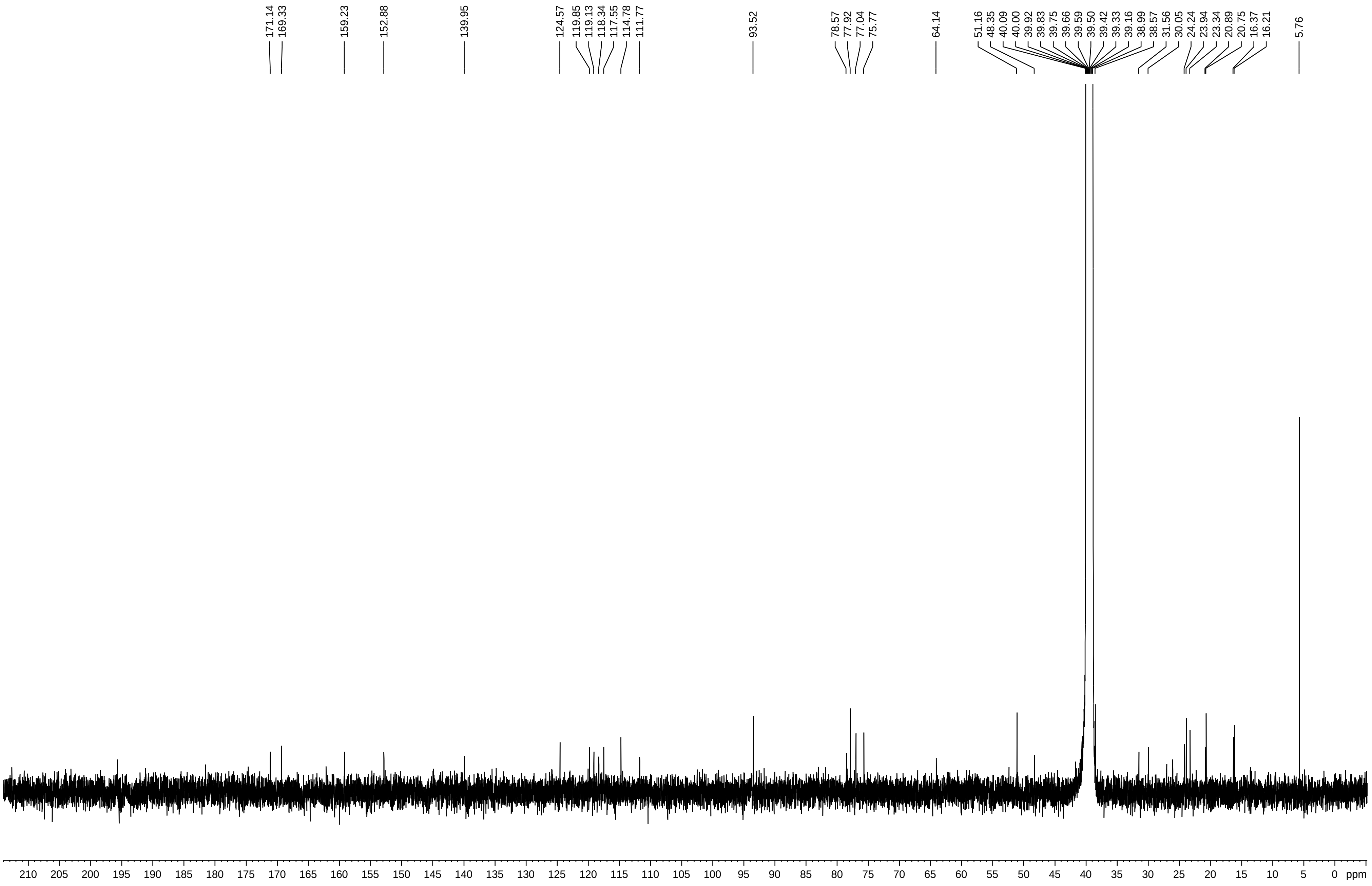

Figure S23. ^{1}H NMR (700 MHz, acetone-d_6) spectrum of 3″-hydroxyterphenyllin (**5**)

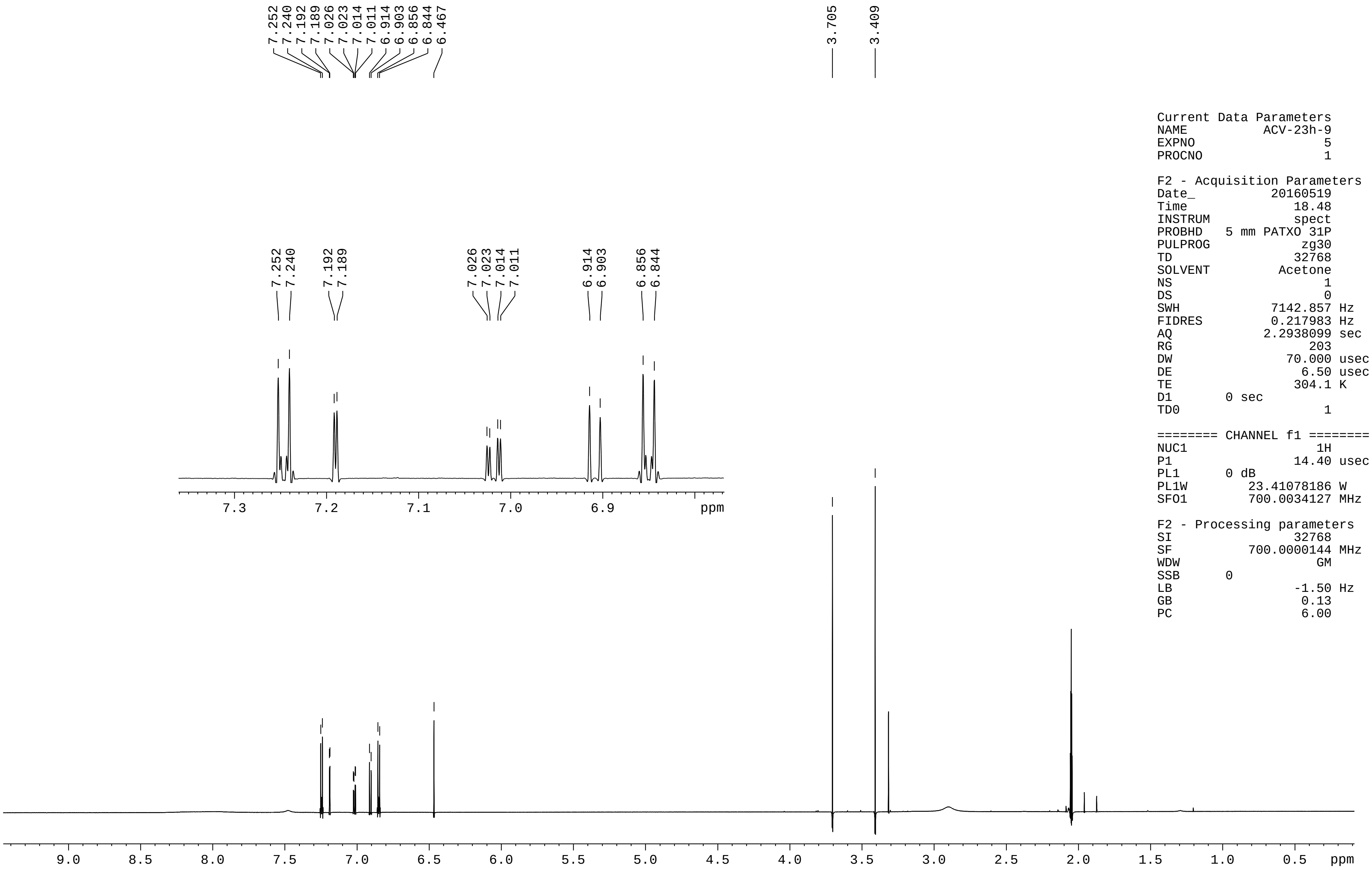

Figure S24. ^{13}C NMR (125 MHz, acetone-d_6) spectrum of 3″-hydroxyterphenyllin (**5**)

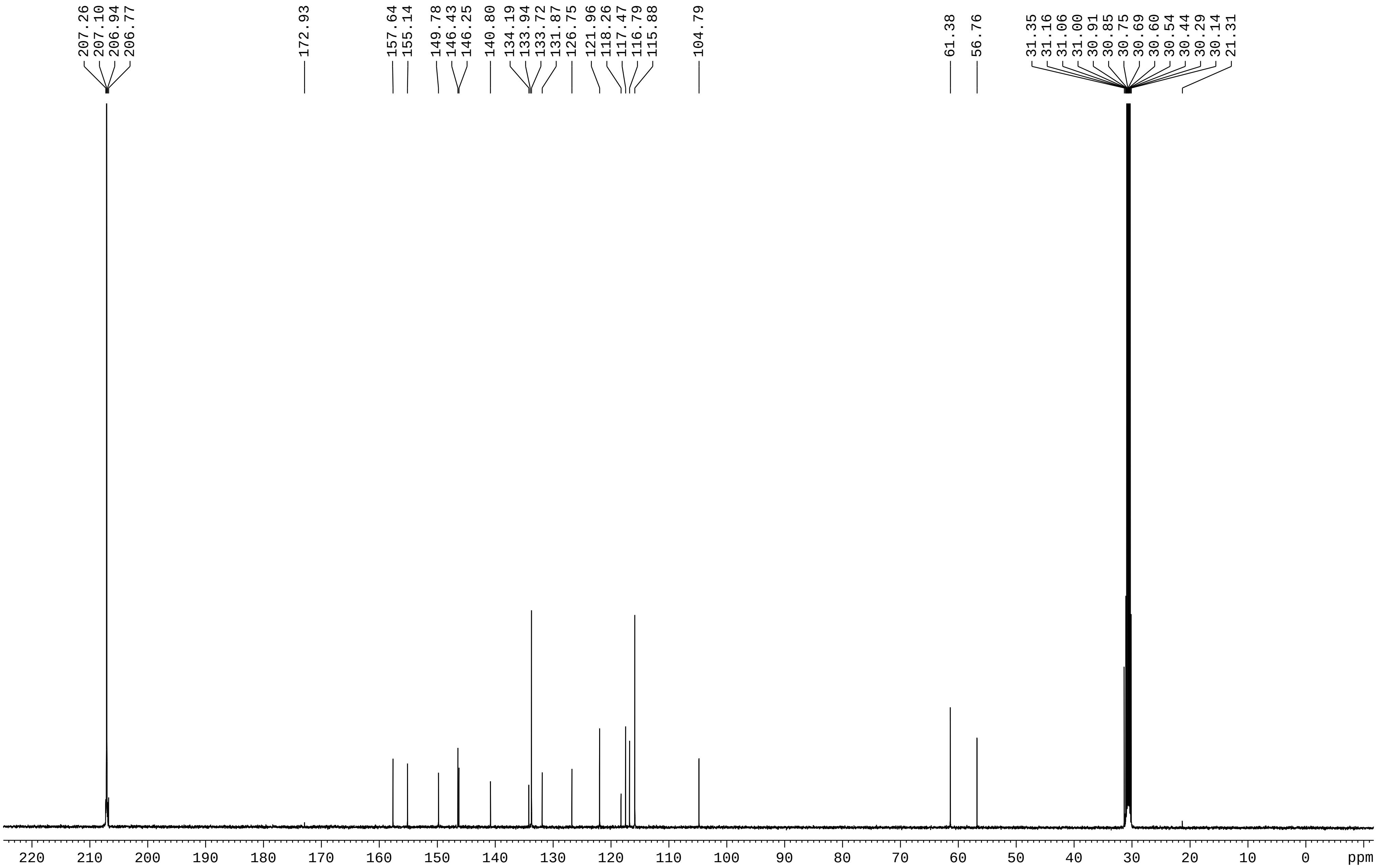

Figure S25. DEPT-135 (125 MHz, acetone-d$_6$) spectrum of 3″-hydroxyterphenyllin (**5**)

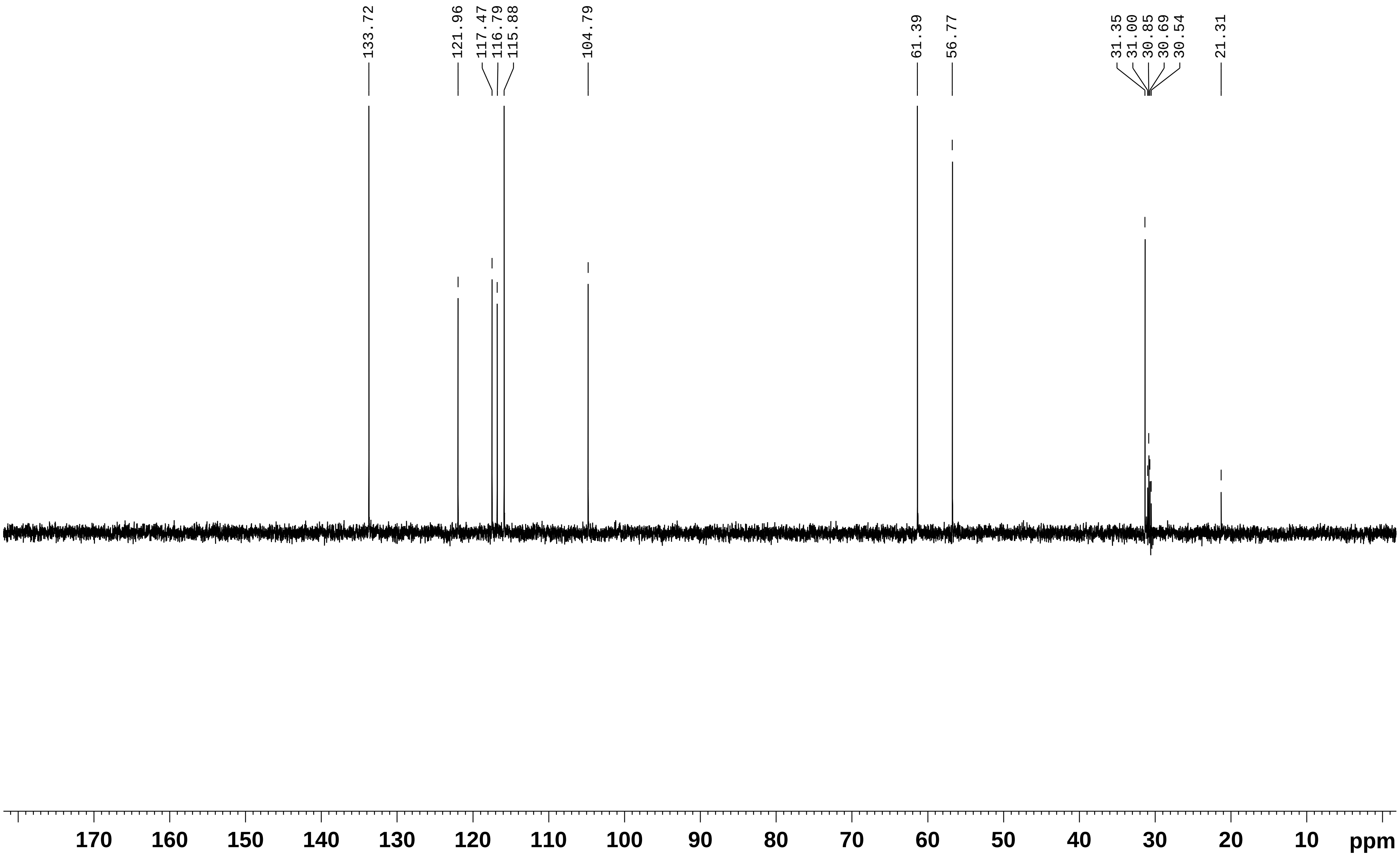

Mar. Drugs 2018, 16, 232; doi:10.3390/md16070232

Figure S26. HSQC (700 MHz, acetone-d_6) spectrum of 3″-hydroxyterphenyllin (**5**)

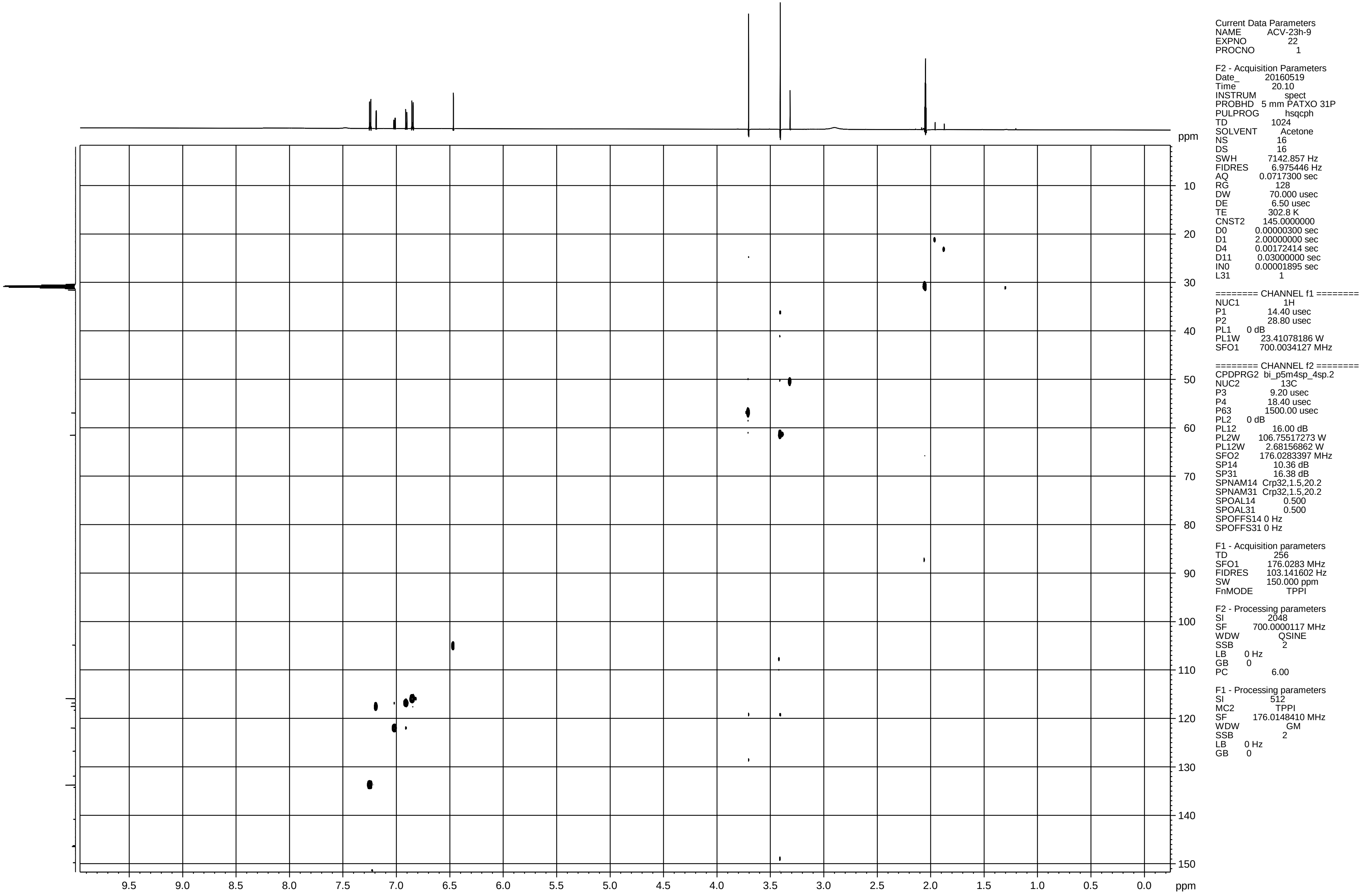

Mar. Drugs 2018, 16, 232; doi:10.3390/md16070232

Figure S27. HMBC (700 MHz, acetone-d$_6$) spectrum of 3″-hydroxyterphenyllin (**5**)

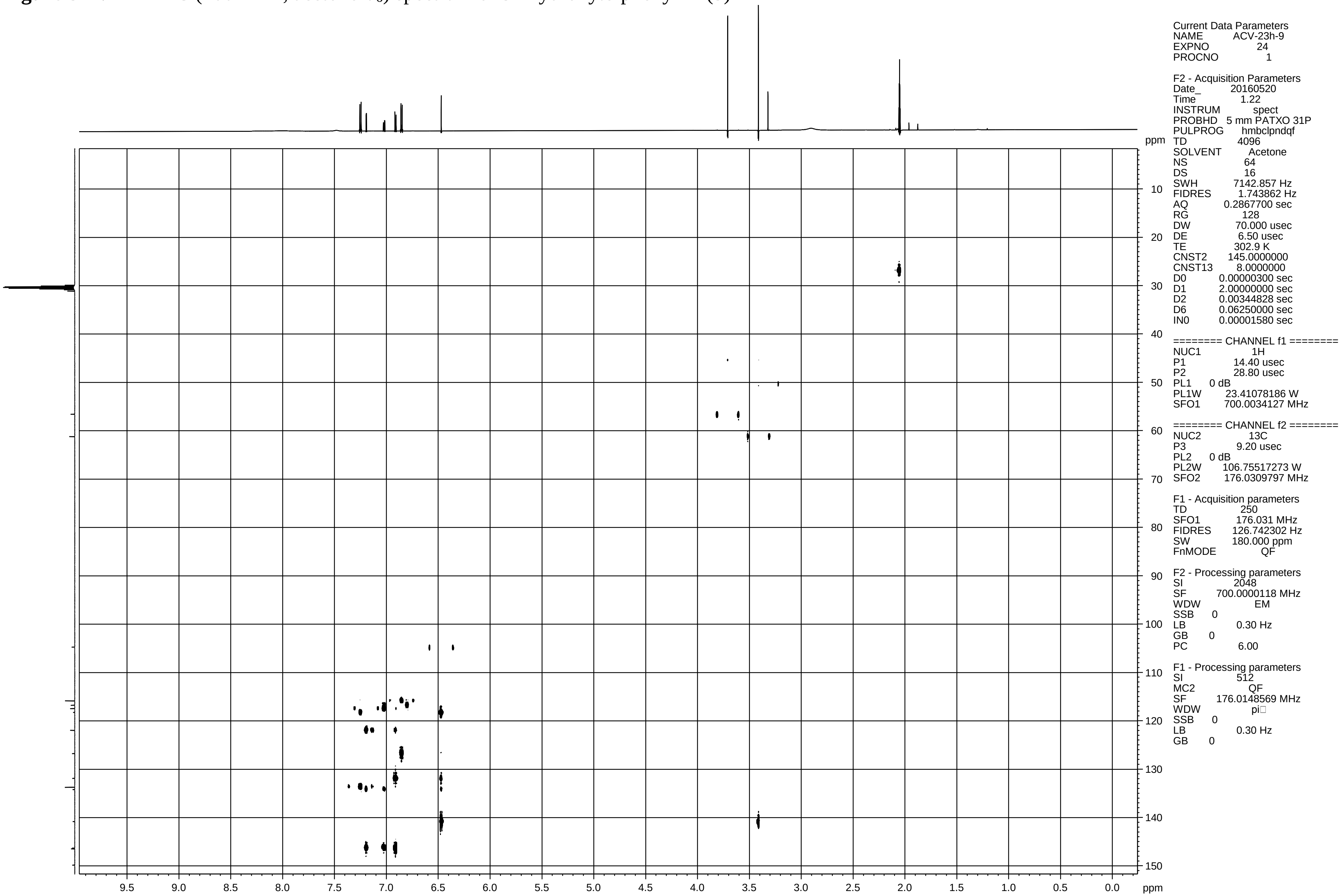

Mar. Drugs 2018, 16, 232; doi:10.3390/md16070232

Figure S28. ROESY (700 MHz, acetone-d$_6$) spectrum of 3″-hydroxyterphenyllin (**5**)

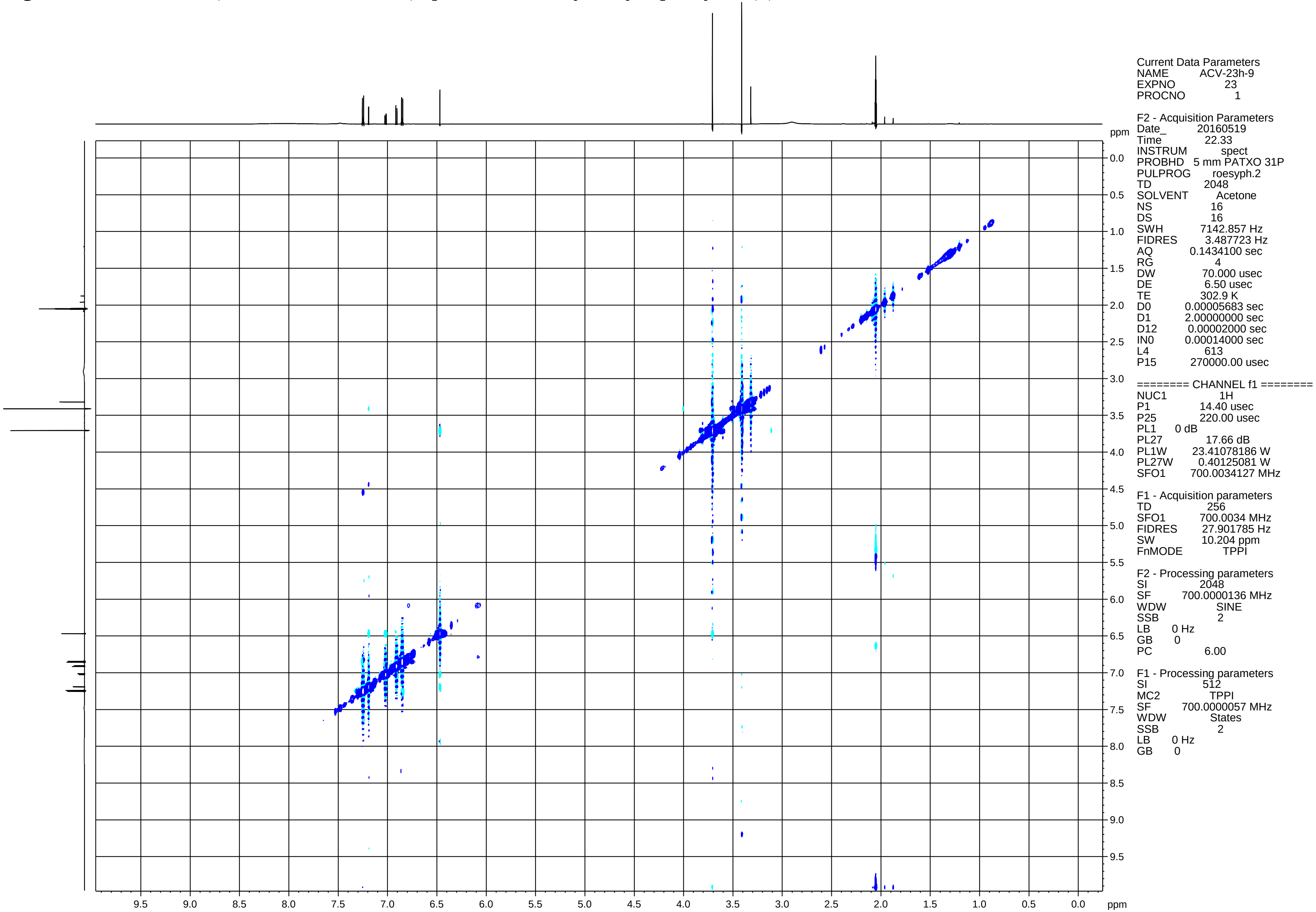

Mar. Drugs 2018, 16, 232; doi:10.3390/md16070232 www.mdpi.com/journal/marinedrugs
